# Sex Differences between Urinary Phthalate Metabolites and Metabolic Syndrome in Adults: A Cross-Sectional Taiwan Biobank Study

**DOI:** 10.3390/ijerph191610458

**Published:** 2022-08-22

**Authors:** Ya-Ling Shih, Chia-Jung Hsieh, Tso-Ying Lee, Pei-Hung Liao, Hao-Ting Wu, Chieh-Yu Liu

**Affiliations:** 1 Department of Nursing, Yuanpei University of Medical Technology, Hsinchu 30015, Taiwan; 2 School of Nursing, College of Nursing, National Taipei University of Nursing and Health Sciences, Taipei 112303, Taiwan; 3 Nursing Research Center of Taipei Medical University Hospital, Taipei 110301, Taiwan; 4 Department of Nursing, Cheng Hsin General Hospital, Taipei 112303, Taiwan; 5 Department of Health Care Management, National Taipei University of Nursing and Health Sciences, Taipei 112303, Taiwan

**Keywords:** phthalates, metabolic syndrome (MetS), endocrine disrupters, sex differences

## Abstract

Background: Phthalates are widely used in consumer products, food packaging, and personal care products, so exposure is widespread. Several studies have investigated the association of phthalate exposure with obesity, insulin resistance, and hypertension. However, little is known about the associations of phthalate exposure with sex, age, and menopausal status in metabolic syndrome (MetS). The purpose of this study was to investigate the association between 11 urinary phthalate metabolite concentrations and metabolic syndrome in adults. Methods: We conducted a cross-sectional analysis of 1337 adults aged 30–70 years from the Taiwan Biobank 2016–2020. Prevalence odds ratios (POR) and 95% confidence intervals (CIs) were calculated using logistic regression and stratified by sex, age, and menopausal status. Results: Participants with MetS comprised 16.38%. Higher concentrations of MEP metabolites were associated with more than two- to three-fold increased odds of MetS in males and males ≥ 50 years (adj. POR Q3 vs. Q1 = 2.13, 95% CI: 1.01, 4.50; *p* = 0.047 and adj. POR Q2 vs. Q1 = 3.11, 95% CI: 0.13, 8.63; *p* = 0.029). When assessed by menopausal status, postmenopausal females with higher ∑DEHP concentrations had more than nine-fold higher odds of MetS compared with postmenopausal females with the lowest ∑DEHP concentrations (adj. POR Q3 vs. Q1 = 9.58, 95% CI: 1.18, 77.75; *p* = 0.034). Conclusions: The findings suggest differential associations between certain phthalate metabolites and MetS by sex, age, and menopausal status.

## 1. Introduction

The Joint Interim Statement definition of metabolic syndrome (MetS) requires that at least three of the following five clinical findings are met: elevated waist circumference, elevated triglycerides, reduced HDL cholesterol, elevated blood pressure, and elevated fasting glucose [1]. MetS raises the risk of type 2 diabetes, cardiovascular disease, and mortality [2,3] and predicts many degenerative diseases in later life [4,5]. It has also been linked to a number of cancers, including breast, pancreatic, colon, and liver [6]. The global prevalence of metabolic syndrome is estimated to be around one-quarter of the world’s population [3]. Moreover, in most countries in the Asia-Pacific region, nearly one in five or more adults are affected by metabolic syndrome, and the prevalence has been increasing over time [7].

According to the American Endocrine Society’s second scientific statement on endocrine-disrupting chemicals (EDCs), based on evidence from animal models, clinical observations, and epidemiological studies, phthalates affect obesity, diabetes, cardiovascular disease, female reproduction, male reproduction, female-hormone-sensitive cancers, prostate cancer, thyroid, and the neurodevelopmental and neuroendocrine systems [8,9,10]. As a class of endocrine-disrupting chemicals, phthalates are the most widely used plasticizers in the world, with an annual consumption of 7.5 million tons. They are found in numerous products, such as vinyl flooring, adhesives, cleaners, lubricants, automotive plastics, children’s toys, textiles, wallpaper, food packaging, and personal hygiene products [11]. They are common in the environment and can enter the human body through the respiratory tract, digestive tract, and skin. The obesity/diabetes panel determined that phthalate exposure was responsible for 53,900 cases of obesity and 20,500 new cases of diabetes in older women each year. The total cost of all conditions likely attributable to EDCs was EUR 191 billion, with phthalate-attributable adult obesity accounting for the second-largest cost driver, at EUR 15.6 billion per year [12]. A food-safety incident in Taiwan in 2011, which revealed that people had been exposed to phthalate-contaminated food for decades, drew public attention to the negative health effects of plasticizers and prompted the government to restrict the use of certain plasticizers. Despite this, a number of studies have found that concentrations of phthalate metabolites in the bodies of the residents of Taiwan are still slightly higher than the global average due to widespread use of plastic bags, plastic wrap, plastic containers, and plastic bottles to package food or beverages [13,14,15,16,17,18].

Several studies have investigated the associations between phthalate exposure and obesity, insulin resistance, and hypertension [8,12,19,20,21]. However, little is known about the association between phthalate metabolites and MetS, and the findings to date have arrived at inconsistent conclusions [22,23,24,25]. Ghosh et al. reported differential associations between phthalate metabolites and MetS by sex and ethnicity. Higher MCOP levels were significantly associated with increased odds of MetS among Caucasian women (POR Q4 vs. Q1 = 1.68, 95% CI: 1.24, 2.29; *p*-trend = 0.001). ΣDEHP metabolites were associated with increased odds of MetS only among Caucasian men (POR Q4 vs. Q1 = 1.54, 95% CI: 1.01, 2.35; *p*-trend = 0.06) [22]. Another study revealed that higher ΣDEHP metabolite concentrations were associated with increased odds of MetS in men (adj. POR for men Q4 vs. Q1 = 2.20; 95% CI: 1.32, 3.68), and the strongest association was between higher concentrations of MBzP and MetS among pre-menopausal women (adj. POR Q4 vs. Q1: 3.88; 95% CI: 1.59, 9.49) [23]. There was a suggestive positive association between intermediate concentrations of MnBP and odds of MetS (POR T2 vs. T1 = 2.66, 95% CI: 0.98–7.24; POR T3 vs. T1 = 2.11, 95% CI: 0.71–6.27) [24]. The three studies mentioned above were all conducted in the United States. However, Shim et al. found that the concentration of mono(2-ethyl-5-hydroxyhexyl phthalate) (MEHHP) was significantly associated with MetS (POR = 1.39) in Korea [25].

The number of studies investigating the association between phthalates and MetS in the Asian population is particularly limited, and only one study has investigated the association between urinary phthalate concentrations and MetS in East Asian (Korean) adults [25]. The objective of this cross-sectional analysis was to investigate the association between 11 urinary phthalate metabolite concentrations and MetS in Taiwan adults using Taiwan Biobank data, for the survey years 2016–2020. 

The purpose of this study was to investigate the associations and significant effects of 11 urinary phthalate metabolites and MetS in adults using data from the Taiwan Biobank collected from 2016 to 2020. The associations of urinary phthalate metabolite concentrations with MetS components in different sexes, as well as women’s menopausal status, were investigated further.

## 2. Methods

### 2.1. Study Design and Data Collection

The Taiwan Biobank (TWB) began collecting data on cancer-free volunteers (150,710 participants) aged 30–70 with questionnaires, self-reported diagnoses, clinical examinations, and tests from 2012, as the baseline enrollment. This cross-sectional study used data from the TWB on 1337 participants aged 30–70 who had their phthalate metabolites measured. To protect the participants, all participants provided informed consent, and all personally identifiable information in the Taiwan Biobank data was encrypted. This study was approved by both Cheng Hsin General Hospital (CHGH-IRB No: (862)110-08), and the Ethic Governance Committee of Taiwan Biobank (TWBR11007-06), and it was conducted following the Declaration of Helsinki guidelines on research involving human subjects.

### 2.2. Measures

#### 2.2.1. Study Population

The initial analysis population included 1653 participants who had phthalatemetabolite data during the 2016–2020 survey. The final analysis sample consisted of 1337 adults over 30 years of age, excluding those with missing data on questionnaires (*n* = 360) and blood tests (*n* = 1).

Physical examinations and sample collection were conducted by qualified and trained professionals with medical backgrounds, who collected data on height, weight, body fat, waist circumference, blood pressure, glycated hemoglobin, serum glucose, serum triglycerides, and serum high-density lipoprotein. Professionals conducted questionnaire interviews and compiled records, including basic personal information, age, education level, marital status, working status, smoking status, alcohol consumption status, betel nut consumption status, regular exercise, weight control, and menopause status. Moreover, education level (illiterate, literate, elementary school, junior high school, senior high school, undergraduate, and graduate school or above), marital status (single, married, separated/divorced, and widowed), working status (yes/no), alcohol consumption (no, ever/stop, and yes), smoking status (never smoker, former smoker, and current smoker), betel nuts consumption (no, occasional or socializing, and yes), regular exercise (yes/no), weight control (yes/no), and menopausal status (premenopausal and postmenopausal) were collected.

#### 2.2.2. Measurement of Phthalate Metabolites

To 1 mL of urine, 10 μL of the isotope mixture was added, followed by 250 μL of ammonium acetate (1 M, pH 6.5) and 3 μL of beta-glucuronidase. The samples were mixed for 5–10 s before being immersed in a 37 °C water bath for 90 min to convert each phthalate metabolite to its unbound form. The urine sample was then allowed to stand at room temperature for 10 min after the water bath, and 2 mL of phosphate buffer (PB) was added to acidify to pH = 2 and mixed for 5–10 s before being collected for solid-phase extraction (SPE) purification. Finally, phthalate metabolites in spot urine samples were determined using high-performance liquid chromatography–electrospray ionization tandem mass spectrometry (HPLC-ESI-MS/MS).

A total of 11 phthalate metabolites were measured during the 2016–2020 period, including mono-2-ethylhexyl phthalate (MEHP), mono-(2-ethyl-5-oxohexyl) phthalate (MEOHP), mono-(2-ethyl-5-hydroxyhexyl) phthalate (MEHHP), metabolites of mono-(2-ethyl-5-carboxypentyl) phthalate (MECPP), Mono(2-carboxymethylhexyl) phthalate (MCMHP), mono-benzyl phthalate (MBzP), mono-ethyl phthalate (MEP), mono-isobutyl phthalate (MiBP),mono-n-butyl phthalate (MnBP), Mono-methyl phthalate (MMP), and Mono-isooctyl phthalate (MiNP). In addition to the analysis of the individual metabolites, the following summary measures were generated and analyzed: high molecular weight (ΣHMW) metabolites (MEHP + MEOHP + MEHHP + MBzP + MiNP), low molecular weight (ΣLMW) metabolites (MEP + MiBP + MnBP + MMP), DEHP (ΣDEHP) metabolites (MEHP + MEOHP + MEHHP + MECPP + MCMHP), and DBP (ΣDBP) metabolites (MiBP + MnBP). The log10 values were the result of determining a wide range of phthalate metabolites (from 0.08 to 4969.05 μg/L creatinine). According to previous studies, the measured concentrations below the limit of detection (LOD) were replaced by the LOD divided by the square root of two [17,22,23,24].

#### 2.2.3. Metabolic Syndrome

Slightly differing from the National Cholesterol Education Program (NCEP) Adult Treatment Panel III (ATP III) definition [1,26], metabolic syndrome (MetS) was defined according to the guidelines of the Health Administration, Ministry of Health and Welfare, Taiwan [27]. Participants were classified as having MetS if they met at least three of the following five criteria: waist circumference ≥90 cm (men) or ≥80 cm (women), blood pressure ≥130/85 mmHg or treatment for hypertension, fasting blood glucose ≥100 mg/dL or treatment for diabetes, fasting serum triglycerides ≥150 mg/dL, and high-density lipid (HDL) cholesterol <40 mg/dL (men) or <50 mg/dL (women). The mean of two or three consecutive blood pressure measurements, or whether current blood pressure medication use was indicated in the interview questionnaire, was used to determine hypertension (yes/no). Hyperglycemia (yes/no) was defined by plasma glucose levels or the presence of current insulin/diabetic medication use on the interview questionnaire.

### 2.3. Statistical Analysis

The distribution of demographic characteristics is presented as means ± standard error of the mean (SE) or number (%), and we compared male and female characteristics using the Rao–Scott chi-squared (χ^2^) test or independent samples *t*-test, depending on the type of variable. Population characteristics of all participants were examined, including metabolic syndrome status and sex. We applied the creatinine correction procedure of O’Brien et al. to adjust for urine dilution [28]. To correct for right skewness, urinary phthalate metabolite concentrations were log-transformed (log-10-transformed) before further analysis. The distributions of urinary phthalate metabolite concentrations were presented as means with the interquartile range (IQR). Furthermore, because the purpose of this study is to examine the dose–response relationship between phthalate metabolites and metabolic syndrome, as well as to comprehend and compare the state of metabolic syndrome at various cut points (concentrations). This study uses quartiles in the same way as previous studies [17,22,23,25].

Multiple logistic regression analysis with MetS status as a dependent variable was used to adjust covariates that were significant in the univariate analysis. Since the urinary phthalate metabolite concentrations were non-normally distributed, we used estimates of interquartile (IQR) concentrations after they were log-10-transformed in all regression models.

Following that, logistic regression was used to estimate prevalence odds ratios (PORs) and 95% confidence intervals (CIs) as measures of the association between phthalate metabolites and metabolic syndrome. Since the time of urine collection may affect the measured urinary phthalate metabolite concentrations, Model 1 was adjusted for urinary creatinine. Since MetS in this study was significantly different with age (*p* < 0.001), sex, education level, and working status (*p* = 0.001, 0.003, and 0.002), which was also consistent with many other studies [7,22,23,24,29], Model 2 was adjusted for potential influencing factors, including age (continuous), sex (male and female), education level (illiterate, literate, elementary, junior, senior, undergraduate, and graduate), and working status (yes, no, and unknown). To retain the observations with missing values in the logistic regression models, an unknown category was included (refusals, do not know, and missing responses) [22]. Furthermore, we investigated the fully adjusted relationship between the quartiles of each phthalate metabolite and each component of MetS. Given that the pathophysiology of MetS differs by sex [22,23,24,29], similar statistical modeling was performed in males and females separately.

Age [7,29] and menopausal status [23] are both important predictors of metabolic syndrome. To assess if the associations between phthalates and metabolic syndrome differed by age, we evaluated the associations in males and females classified as <50 and ≥50 years of age. We assessed the association between phthalates and metabolic syndrome in premenopausal and postmenopausal women to explore if there were any differences in menopausal status. Bonferroni corrections were not used for multiple comparisons.

All statistical analyses were conducted in IBM SPSS Statistics for Windows, Version 26.0 (IBM Corporation, Armonk, NY, USA). A *p*-value < 0.05 was considered to be statistically significant.

## 3. Results

### 3.1. Participants’ Demographics Based on MetS Status

The study population consisted of 1337 adult participants, 693 (51.83%) males and 644 (48.17%) females. The prevalence of metabolic syndrome was 16.40% (*n* = 219). The people with MetS were more likely to be female (*p* < 0.001) and older (*p* < 0.001) and have a higher body mass index (*p* < 0.001), a higher body fat ratio (*p* < 0.001), a higher glycated hemoglobin (*p* < 0.001), a high waist circumference (*p* < 0.001), higher blood pressure (*p* < 0.001), higher serum glucose (*p* < 0.001), higher triglycerides (*p* < 0.001), and lower HDL (*p* < 0.001). In addition, there were significant differences in education level and working status (*p* = 0.003 and *p* = 0.002). When the results were further stratified by sex (male vs. female) and MetS status (MetS vs. No MetS), the same results were obtained. Males were also significantly different when it came to alcohol and betel nut consumption (*p* = 0.035 and *p* = 0.014, respectively). However, in the overall study population, smoking (*p* = 0.383), regular exercise (*p* = 0.894), and weight control (*p* = 0.970) were not significantly different (Table 1).

Furthermore, the prevalence of MetS was 12.6% for females aged <50 years and 26.0% for females aged ≥50 years. MetS was found in 8.7% of premenopausal females and 11.2% of postmenopausal females.

### 3.2. Differences in MetS Status and Urinary Phthalate Metabolite Concentrations

The highest geometric means (GMs) of urinary phthalate metabolite concentrations (μg/L) in the overall population were MnBP (GM = 21.28, 95% CI: 21.14, 21.41), MECPP (GM = 18.58, 95% CI: 18.47, 18.70), MEHHP (GM = 12.83, 95% CI: 12.69, 12.97), and MEP (GM = 12.55, 95% CI: 12.33, 12.76), while that of MiNP was the lowest (GM = 0.43, 95% CI: 0.32, 0.54). When stratified by MetS, urinary phthalate metabolite concentrations appeared to be slightly higher in the MetS group than in the non-MetS group. However, we found that only MEP concentrations were significantly different in the MetS group and the non-MetS group (*p* = 0.026) (Table 2). When stratified by sex, females had slightly higher concentrations of urinary phthalate metabolites than males, except for MiBP.

### 3.3. Multiple Logistic Regression Analysis between MetS Status and Urinary Phthalate Metabolite Concentrations

We evaluated the prevalence odds ratios (PORs) and 95% Cis for MetS status and compared them to the urinary phthalate metabolite concentrations for each item for males and females (Table 3). There were statistically significant crude associations between ΣLMW, and MEP with MetS prevalence. Across the study population, there was a slightly positive association between ΣLMW and MetS (POR Q2 vs. Q1 = 1.97, 95% CI: 1.30, 2.98; *p* = 0.001). After full adjustment, the odds of developing MetS increased by approximately 65% in those with higher ΣLMW concentrations, compared with the lowest concentration (Q1) (adj. POR Q2 vs. Q1 = 1.65, 95% CI: 1.02, 2.66; *p* = 0.042). Conversely, higher MiBP levels were inversely associated with the odds of MetS prevalence (adj. POR Q2 vs. Q1 = 0.60, 95% CI: 0.37, 0.96; *p* = 0.035). Furthermore, given that MEP and ΣLMW are more than moderately associated, a similar association between ΣLMW and MetS was found when both variables were simultaneously adjusted in our model, but the association between MEP and MetS was attenuated and became less significant (adj. POR Q2 vs. Q1 = 1.65, 95% CI: 0.98, 2.79; *p* = 0.06).

On the other hand, the association between MEP and MetS, although not significant, varied by sex: higher concentrations of MEP were associated with more than two-fold increased odds of developing MetS in males (adj. POR Q2 and Q1 = 2.03, 95% CI: 0.93, 4.42; *p* = 0.074 and adj. POR Q3 vs. Q1 = 2.13, 95% CI: 1.01, 4.50; *p* = 0.047). In contrast, no clear association between MEP and MetS was observed in females. Furthermore, the concentration of MBzP was inversely associated with MetS (POR Q2 vs. Q1 = 0.59, 95% CI: 0.39, 0.91; *p* = 0.017), but the association weakened and became less significant after adjustment (adj. POR Q2 vs. Q1 = 0.65, 95% CI: 0.40, 1.04; *p* = 0.07). Furthermore, higher MBzP concentrations in females were associated with fewer MetS morbidity (adj. POR Q2 vs. Q1 = 0.50, 95% CI: 0.26, 0.96; *p* = 0.002). The findings revealed that, while the association between ΣDEHP and MetS was not statistically significant, it differed by sex: higher ΣDEHP concentrations in men were inversely associated with MetS morbidity (adj. POR Q2 vs. Q1 = 0.48, 95% CI: 0.23, 0.98; *p* = 0.037), whereas no such relationship was observed in women. Moreover, ΣLMW was positively associated with MetS in females (POR Q2 vs. Q1 = 1.87, 95% CI: 0.04, 3.36; *p* = 0.037), but the association between ΣLMW and MetS weakened and became insignificant after adjustment. ∑LMW and MEP had non-monotonic associations with prevalent MetS. In fact, the second-exposure quartile appeared to have the strongest association with these phthalates.

### 3.4. Associations between Phthalate Metabolite Quartiles and MetS Components

In the study population, 44.95% had high waist circumference, 28.50% had low HDL, 23.41% had hyperglycemia, 23.04% had high blood pressure, and 19.97% had elevated triglycerides. High waist circumference was the most prevalent individual MetS component, and it was also more prevalent in women (95.15%) than in men (64.22%). We also found that the phthalate metabolites MCMHP, MEP, MnBP, and MiNP were positively associated with individual components of MetS, though some of the associations differed by sex (Table 4, Supplemental Table S1). In the overall population, we found that the highest quartile of MCMHP was positively associated with hyperglycemia (POR Q4 vs. Q1 = 1.72, 95% CI: 1.04, 2.84; *p* = 0.036), and MEP was also positively associated with hyperglycemia (POR Q2 vs. Q1 = 1.74, 95% CI: 1.06, 2.85; *p* = 0.027 and POR Q4 vs. Q1 = 1.73, 95% CI: 1.07, 2.80; *p* = 0.026). Higher concentrations of these two correlated with higher odds of hyperglycemia by approximately 72% to 74%. Higher MnBP was positively associated with high blood pressure (POR Q2 vs. Q1 = 1.57, 95% CI: 1.01, 2.42; *p* = 0.045) and elevated triglycerides (POR Q3 vs. Q1 = 1.54, 95% CI: 1.01, 2.36; *p* = 0.044). Furthermore, higher concentrations of MiNPs were associated with more than three-fold higher odds of developing high triglycerides (POR Q3 vs. Q1 = 3.68, 95% CI: 1.45, 9.35; *p* = 0.006). In contrast, MEHP, MEHHP, MBzP, MiBP, and ΣHMW were inversely associated with each component of MetS.

In males, positive associations were found between phthalate metabolites and various components of MetS, including higher ΣDBP associated with more than two-fold increased odds of developing low HDL (POR Q4 vs. Q1 = 2.31, 95% CI: 1.05, 5.08; *p* = 0.037). Higher MEP was also associated with more than two-fold increased odds of developing hyperglycemia (POR Q2 vs. Q1 = 2.23, 95% CI: 1.19, 4.16; *p* = 0.012), and there was also a more than two-fold association with increased odds of elevated triglycerides (POR Q2 vs. Q1 = 2.04, 95% CI: 1.13, 3.71; *p* = 0.019). Moreover, higher ΣDBP was associated with odds of elevated triglycerides, an increase of about 88% (POR Q2 vs. Q1 = 1.88, 95% CI: 1.06, 3.34; *p* = 0.030). Higher concentrations of MCMHP were suggestively positively associated with high waist circumference (POR Q3 vs. Q1 = 1.71, 95% CI: 1.00, 2.92; *p* = 0.050).

Although only MiNP was found to be positively associated with high triglycerides in females, higher MiNP concentrations were associated with four- to five-fold increased odds of elevated triglycerides. (POR Q3 vs. Q1 = 5.05, 95% CI: 1.75, 14.58; *p* = 0.003 and POR Q4 vs. Q1 = 4.14, 95% CI: 1.03, 16.69; *p* = 0.046). Males showed an inverse association, though it was not statistically significant.

### 3.5. Associations between Quartiles of Phthalate Metabolites and MetS Stratified by Age and Sex

In males, higher concentrations of MEP were associated with more than three-fold higher odds of MetS in older males aged ≥50 years (*n* = 357) (POR Q2 vs. Q1 = 3.11, 95% CI: 0.13, 8.63; *p* = 0.029). However, higher concentrations of ΣDEHP were inversely associated with the odds of developing MetS in older males (POR Q2 vs. Q1 = 0.35, 95% CI: 0.12, 0.98; *p* = 0.045). Higher concentrations of MiBP were found to have a statistically significant negative association in younger males (*n* = 336), as compared with lower concentrations of this phthalate metabolite (POR Q2 vs. Q1 = 0.18, 95% CI: 0.05, 0.75; *p* = 0.018).

In females, higher concentrations of MEOHP (POR Q2 vs. Q1 = 0.32, 95% CI: 0.10, 0.97; *p* = 0.043 and POR Q4 vs. Q1 = 0.31, 95% CI: 0.10, 0.93; *p* = 0.037 ) and MBzP (POR Q2 vs. Q1 = 0.04, 95% CI: 0.01, 0.31; *p* = 0.002, POR Q3 vs. Q1 = 0.37, 95% CI: 0.15, 0.93; *p* = 0.035 and POR Q4 vs. Q1 = 0.31, 95% CI: 0.12, 0.82; *p* = 0.018 ) were inversely associated with the odds of MetS in younger females (*n* = 333). For older females aged ≥ 50 years (*n* = 311), no statistically significant association was found. Only higher ΣDEHP concentrations were found to have a suggestive positive association with the odds of developing MetS (POR Q2 vs. Q1 = 3.97, 95% CI: 0.82, 19.36; *p* = 0.09) (Table 5).

### 3.6. Associations between Quartiles of Phthalate Metabolites and MetS Stratified by Menopause

**Figure 1:**
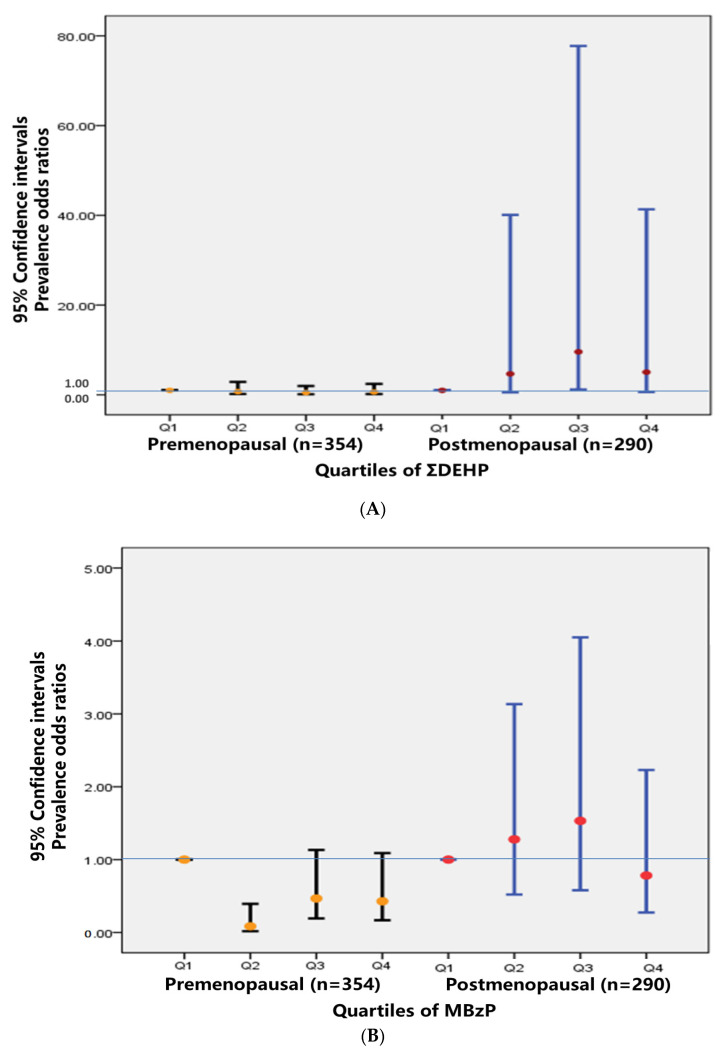
(**A**) Prevalence odds ratios for the association between urinary ∑DEHP metabolites concentrations and metabolic syndrome stratified by menopausal status in females. (**B**) Prevalence odds ratios for the association between urinary MBzP concentrations and metabolic syndrome stratified by menopausal status in females. Prevalence odds ratios adjusted for urinary creatinine, age, education, and working status.

MetS was found to have a strong positive association with ∑DEHP in postmenopausal females (*n* = 290). In fact, the association of this phthalate metabolite was even stronger than in the age- and sex-stratified analyses. Specifically, postmenopausal females with higher ∑DEHP concentrations were nine-fold more likely to be MetS cases than females with the lowest concentrations (POR Q3 and Q1 = 9.58, 95% CI: 1.18, 77.75; *p* = 0.034). ∑DEHP had non monotonic associations with MetS. The strongest association for ∑DEHP appeared to be the third-exposure quartile in postmenopausal females. Higher concentrations of MBzP were found to have a statistically significant negative association in premenopausal females, as compared with lower concentrations of this phthalate metabolite (POR Q2 and Q1 = 0.08, 95 percent CI: 0.02, 0.39; *p* = 0.002) (Figure 1, Supplemental Table S2).

## 4. Discussion

To the best of the authors’ knowledge, this is the first study to evaluate the association between phthalate metabolites and MetS in Taiwan. The purpose of this study was to investigate the association between urinary phthalate metabolites and metabolic syndrome in adults and to identify important influencing factors. Secondary objectives were to evaluate these differential analyses in relation to sex, MetS components, age, and menopausal status.

In the overall study population, smoking, regular physical activity, and weight control did not differ significantly between individuals with and without MetS. These findings are consistent with previous research that found no difference between smoking status [25,30] and regular exercise [24,25] and MetS. However, several studies [23,29,31] have produced different results. Although some health-harming and health-promoting behaviors did not differ significantly in this study, providing educational resources and advocating for informed public health policies are critical in disease prevention [31].

Significant differences were found in this study between MEP and individuals with or without MetS in the overall study population. This means that people with MetS have higher levels of MEP in their urine. This is consistent with previous research. Adolescents with MetS have slightly higher urinary phthalate metabolite concentrations. The MEP concentration is the most significant difference [24].

After adjusting for various sociodemographic and lifestyle factors in this study, there was still a clear positive association between ∑LMW and MetS: adults with moderate levels of ∑LMW were 60% more likely to develop MetS than those with the lowest levels. The findings of James-Todd et al. differed from those of the current study, which found that higher concentrations of MBzP and ∑DEHP were associated with an increased risk of MetS, as compared with the lowest concentrations in the overall study population. In our study, females had slightly higher concentrations than males. Previous studies have also found that women have higher concentrations of phthalate metabolites than men, possibly due to women’s higher use of personal hygiene products and cosmetics, followed by increased skin sensitivity to phthalate exposure [24,32,33,34], which contradicts another study [22].

The association between phthalate metabolites and MetS odds may differ by sex. Males with higher MEP concentrations had higher odds of developing MetS, while males with moderate concentrations were more than three-fold likely to increase odds of MetS than men with the lowest concentrations, particularly those aged ≥ 50 years, as well as higher odds of developing hyperglycemia and elevated triglycerides. These findings differ from those of previous studies, suggesting that higher levels of ∑DEHP metabolites are associated with increased odds of developing MetS in males [22,23]. Another study found that higher MEP concentrations in Mexican women increased their odds of elevated triglycerides [30]. Higher MEP concentrations were found to be negatively correlated with the likelihood of MetS in black men, according to Ghosh et al. [22].

When the components of metabolic syndrome were examined further, this study discovered that higher MnBP concentrations were associated with high blood pressure and were also significantly associated with elevated triglycerides. MEP and MCMHP were also found to be positively associated with hyperglycemia, and MiNP was positively associated with elevated triglycerides. In contrast to the current study, James-Todd et al. used the 2001–2010 National Health and Nutrition Examination Survey (NHANES) in the United States and found that the metabolite MBzP was significantly associated with high waist circumference and elevated triglycerides and that higher ∑DEHP was associated with increased odds of high waist circumference, elevated triglycerides, high blood pressure, and hyperglycemia [23]. Adjusted MnBP concentrations were associated with increased odds of high waist circumference, elevated triglycerides, high blood pressure, and hyperglycemia in a study investigating phthalate metabolites and MetS in U.S. adolescents [24]. According to the findings of this study, other factors that increase the risk of cardiovascular disease, such as low HDL and high blood pressure, may be associated with higher phthalate exposure, which is consistent with the findings of James-Todd et al. [23]. While these associations differed between males and females, MiNP was associated with elevated triglycerides in females, while MEP was associated with hyperglycemia and elevated triglycerides in males. In contrast to the findings of this study, one study found that MBzP was associated with high waist circumference in males, DEHP was associated with abdominal obesity and hypertension in men, MBzP was associated with abdominal obesity and hyperglycemia in females, and ∑DEHP was associated with higher odds of elevated triglycerides in females [23]. Another study, which investigated whether urinary phthalate metabolite concentrations in Mexican women in 2008 were associated with MetS and its components in midlife 9 years later, showed that ΣDBP, MBzP, and MEP were associated with increased odds of elevated triglycerides [22]. High MnBP levels in adolescent males were associated with higher odds of elevated triglycerides and lower HDL [24]. As a result, the associations between phthalate metabolites and MetS constituents varied according to sex, ethnicity, and age.

The analyses in this study were stratified by a number of influencing factors, including sex, age, and menopausal status, because phthalate metabolite concentrations and MetS morbidity rates can vary depending on these factors [11,22,23,24,35]. The association was stronger among postmenopausal females and males aged ≥ 50 years. These differences could be due to hormonal differences or the effects of aging. We discovered no age-related relationship in females. When stratified by menopausal status, however, higher ∑DEHP concentrations were associated with up to nine-fold greater odds of MetS in postmenopausal females compared with the lowest concentrations. A study conducted in the United States found that higher ∑DEHP concentrations in female participants were associated with a younger age at menopause [36]. Díaz Santana et al. investigated the association of urinary phthalate metabolite concentrations with body-weight change in postmenopausal women (*n* = 997) and found that the highest quartile of ΣDEHP were two times more likely to be overweight (OR 2.72, 95% CI 1.57–4.72) and three times more likely to be obese (OR 3.29, 95% CI 1.80–6.03), compared to those in the lowest quartile [37]. ΣDBP and ΣDEHP have been shown to inhibit regular activity of T3 [38], which could affect weight gain because phthalates disrupt the thyroid hormone system. Another study found that females with infertility, recurrent miscarriages, and tubal factors of endocrine dysfunction had higher ∑DEHP concentrations [39]. The results of this study differ from those of previous studies, which indicated that in premenopausal women, the strongest association was with higher concentrations of MBzP [27]. According to Ghosh et al., the association between ∑DEHP and increased odds of MetS was only observed in white males, with no significant pattern or dose–response relationship of ∑DEHP observed in females, either overall or by ethnicity [22]. Phthalates may also influence menopausal age, which influences disease in postmenopausal women. Future research would be needed to elucidate these associations so as to better understand these potential sex, age, and menopausal-status differences.

DEHP is one of the most commonly used endocrine disruptors [40,41]. ∑DEHP is present in a multitude of common sources of exposure: medical pipes containing polyvinyl chloride (PVC), blood bags, medical equipment, food contamination, food-packaging bags, plastic toys, wall coatings, tablecloths, vinyl floor tiles, furniture cushions, shower curtains, plastic water pipes, swimming pool dividers, raincoats, diapers, dolls, some toys, shoes, vehicle cushions, car roofs, photographic film, plastic wrapping paper, wire and cable wrapping [42], and even indoor air and dust [43]. Thus, it is the endocrine disruptor of greatest concern. Chen et al. proposed that washing hands and drinking less often from plastic cups are the most effective strategies for reducing phthalate metabolites in urine for ∑DEHP [13]. Since phthalates are still used in many consumer products and are being replaced by similar chemicals, future studies and interventions must continue, so as to develop a better understanding about and reduce the impact on health by phthalates.

This study has several limitations. Since non-probability sampling was used for the participants of the Taiwan Biobank, sample-selection bias was possible. Furthermore, due to the cross-sectional study design, we were unable to infer causality and instead emphasized the possibility of reverse causality. MetS in adults may occur prior to phthalate exposure. Since phthalates are non-persistent chemicals, excretion peaks around 4 h after exposure, and they can be completely eliminated within 24 h [44]. Therefore, urinary metabolite concentrations only reflect exposure that occurred ≤1 day prior to urine sample collection and may not reflect long-term exposure. MetS, on the other hand, may take years or decades to manifest, and the short half-lives of these chemicals may be meaningful only if exposure to higher concentrations of phthalates is consistent over time. In addition, phthalates are known to interact with estrogen [45,46,47,48]. Therefore, when investigating these associations, we cannot rule out the role of estrogen. Future longitudinal studies will need to evaluate these chemicals with repeated measurements at multiple time points to determine their association with MetS and its constituents, in addition to collecting steroid hormones to confirm menopausal status. We hope to increase the number of participants in the future to predict the relationship between phthalate metabolites and MetS. Additionally, the quartiles of phthalate metabolites do not imply clinical significance and may not be the threshold for effect. Furthermore, we did not use a more robust cut-off (e.g., false-discovery rate or Bonferroni) to adjust for multiple testing, due to the exploratory nature of this study, so the results could be coincidental. Despite these limitations, this exploratory analysis supports the need for further research to better understand the association between urinary phthalate and MetS in adults.

This research has several strengths. First, to ensure the validity of data, the Taiwan Biobank employs extensive quality control and assurance procedures. Second, we explored the associations between 11 phthalate metabolites and MetS as well as summary measures based on their molecular weight. After adjusting for several confounding factors, we founded an association between urinary MEP and ∑DEHP and MetS in adult participants. Lastly, we assessed sex differences and women’s menopausal status, which is important given the endocrine disrupting properties of phthalates.

## 5. Conclusions

In this cross-sectional study, we found an association between phthalate metabolite concentrations and MetS morbidity by sex, age, and menopausal status. Phthalates are widely used in consumer products, food packaging, and personal hygiene products, so exposure is widespread. Since this was a cross-sectional study, it can generate only causal hypotheses; further case-control studies and prospective studies will be required for confirmation of these emerging hypotheses. Moreover, in addition to further evaluating the role of these metabolites and the risk of MetS, it is recommended that the public’s health literacy about phthalates be improved and that relevant nursing care and health education interventions be provided to help the public reduce its exposure to and use of phthalates. It is also important to understand the long-term effects of these intrinsic disruptors on health and environmental ecology.

## Figures and Tables

**Table 1:** Overall population characteristics and population characteristics stratified by sex and metabolic syndrome status among adults aged 30–70 years (*n* = 1337).

	Total *(n* = 1337)		*p*	Males (*n* = 693)		*p*	Females (*n* = 644)		*p*
	MetS	No MetS		MetS	No MetS		MetS	No MetS	
	(*n* = 219)	(*n* = 1118)	<0.001	(*n* = 91)	(*n* = 602)		(*n* = 128)	(*n* = 516)	
	N (%)								
Age	219 (16.40)	1118 (83.60)	<0.001	91 (13.10)	602 (86.90)	0.077	128 (19.90)	516 (80.10)	<0.001
30–39	44 (3.30)	304 (22.70)		20 (2.90)	160 (23.10)		24 (3.70)	144 (22.40)	
40–49	36 (2.70)	285 (21.30)		13 (1.90)	143 (20.60)		23 (3.60)	142 (22.00)	
50–59	74 (5.50)	302 (22.60)		31 (4.50)	161 (23.20)		43 (6.70)	141 (21.90)	
60–70	65 (4.90)	227 (17.00)		27 (3.90)	138 (19.90)		38 (5.90)	89 (13.80)	
Education level	219 (16.40)	1118 (83.60)	0.003	91 (13.10)	602 (86.90)	0.013	128 (19.90)	516 (80.10)	0.034
Illiterate	1 (0.10)	1 (0.10)		0 (0.00)	0 (0.00)		1 (0.20)	1 (0.20)	
Literate	1 (0.11)	1 (0.11)		1 (1.10)	0 (0.00)		0 (0.00)	1 (0.20)	
Elementary	13 (1.00)	46 (3.40)		1 (1.10)	22 (3.20)		12 (1.90)	24 (3.70)	
Junior	15 (1.10)	76 (5.70)		5 (0.70)	37 (5.30)		10 (1.60)	39 (6.10)	
Senior	71 (5.30)	318 (23.80)		25 (3.60)	146 (21.10)		46 (7.20)	172 (26.80)	
Undergraduate	112 (8.30)	548 (41.00)		54 (7.80)	309 (44.60)		58 (8.90)	239 (37.10)	
Graduate	6 (0.40)	128 (9.60)		5 (0.70)	88 (12.70)		1 (0.20)	40 (6.20)	
Marriage	219 (16.40)	1118 (83.60)	0.132	91 (13.20)	602 (86.80)	0.704	128 (19.90)	516 (80.10)	0.043
Single	25 (1.90)	161 (12.10)		10 (1.40)	77 (11.10)		15 (2.30)	84 (13.10)	
Married	170 (12.70)	836 (62.50)		74 (10.70)	495 (71.40)		96 (14.90)	341 (52.90)	
Separated/Divorced	11 (0.80)	82 (6.10)		5 (0.70)	23 (3.30)		6 (0.90)	59 (9.20)	
Widowed	13 (1.00)	39 (2.90)		2 (0.30)	7 (1.00)		11 (1.70)	32 (5.00)	
Working Status	161 (16.93)	790 (83.07)	0.002	67 (14.00)	411 (86.00)	0.048	94 (19.90)	379 (80.10)	0.033
Yes	87 (9.15)	528 (55.52)		39 (8.20)	289 (60.50)		48 (10.10)	239 (50.00)	
No	74 (7.78)	262 (27.55)		28 (5.90)	122 (25.50)		46 (9.70)	140 (29.60)	
Alcohol Consumption Status	219 (16.40)	1118 (83.60)	0.327	91 (13.20)	602 (86.80)	0.035	128 (19.90)	516 (80.10)	0.700
No	189 (14.10)	1000 (74.80)		66 (9.50)	499 (72.00)		123 (19.10)	501 (77.80)	
Ever, Stop drinking	10 (0.70)	33 (2.50)		9 (1.30)	28 (4.00)		1 (0.20)	5 (0.80)	
Yes	20 (1.50)	85 (6.40)		16 (2.30)	75 (10.80)		4 (0.60)	10 (1.60)	
Smoking Status	219 (16.40)	1118 (83.60)	0.383	91 (13.20)	602 (86.80)	0.483	128 (19.90)	516 (80.10)	0.79
Never Smoker	173 (12.90)	847 (63.40)		55 (7.90)	363 (52.40)		118 (18.30)	484 (75.20)	
Former Smoker	28 (2.10)	144 (10.80)		23 (3.30)	127 (18.30)		5 (0.80)	17 (2.60)	
Current Smoker	18 (1.30)	127 (9.50)		13 (1.90)	112 (16.20)		5 (0.80)	15 (2.30)	
Betel Nut Consumption	219 (16.40)	1118 (83.60)	0.092	91 (13.20)	602 (86.80)	0.014	128 (19.90)	516 (80.10)	0.618
No	212 (15.90)	1098 (82.10)		84 (12.10)	583 (84.10)		128 (19.90)	515 (80.00)	
Occasional or Socializing	6 (0.40)	11 (0.80)		6 (0.90)	10 (1.40)		0 (0.00)	1 (0.20)	
Yes (Every Day)	1 (0.10)	9 (0.70)		1 (0.10)	9 (1.30)		0 (0.00)	0 (0.00)	
Regular Exercise	219 (16.40)	1118 (83.60)	0.894	91 (13.20)	602 (86.80)	0.147	128 (19.90)	516 (80.10)	0.078
Yes	94 (7.00)	474 (35.50)		35 (5.10)	280 (40.50)		59 (9.20)	194 (30.10)	
No	125 (9.40)	644 (48.10)		56 (8.10)	322 (46.40)		69 (10.70)	322 (50.00)	
Weight Control	219 (16.4)	1118 (83.6)	0.970	91 (13.20)	602 (86.80)	0.710	128 (19.90)	516 (80.10)	0.468
Yes	154 (11.50)	721 (53.90)		68 (9.80)	392 (56.60)		86 (13.40)	329 (51.10)	
No	65 (4.90)	397 (29.70)		23 (13.10)	210 (30.30)		42 (6.50)	187 (29.00)	
Menopausal Status	-	-	-	-	-	-	128 (19.90)	516 (80.10)	0.004
Premenopausal	-	-		-	-		56 (8.70)	298 (46.30)	
Postmenopausal	-	-		-	-		72 (11.20)	218 (33.90)	

**Table 2:** Urinary phthalate metabolites by metabolic syndrome status.

	Metabolic Syndrome			No Metabolic Syndrome			*p*
	(*n* = 219)			(*n* = 1118)			
	Geometric Mean (95% CI)			Geometric Mean (95% CI)			
MEHP	10.98	(10.82,	11.15)	9.43	(9.27,	9.59)	0.058
MEOHP	8.02	(7.91,	8.14)	8.00	(7.88,	8.12)	0.961
MEHHP	12.87	(12.75,	12.99)	12.83	(12.70,	12.95)	0.960
MECPP	18.60	(18.49,	18.71)	18.58	(18.46,	18.69)	0.979
MCMHP	4.50	(4.32,	4.67)	4.02	(3.83,	4.20)	0.214
MBzP	1.13	(0.98,	1.28)	1.15	(0.99,	1.30)	0.875
MEP	15.28	(15.04,	15.52)	12.07	(11.85,	12.29)	0.026
MiBP	8.25	(8.12,	8.38)	9.18	(9.04,	9.33)	0.141
MnBP	20.85	(20.72,	20.98)	21.36	(21.23,	21.49)	0.714
MMP	2.28	(2.13,	2.44)	2.07	(1.91,	2.23)	0.223
MiNP	0.45	(0.31,	0.58)	0.43	(0.30,	0.56)	0.539
ΣHMW	37.91	(37.80,	38.03)	36.14	(36.03,	36.26)	0.390
ΣLMW	62.22	(62.08,	62.35)	58.28	(58.15,	58.41)	0.319
ΣDEHP	61.97	(61.86,	62.08)	59.83	(59.72,	59.94)	0.508
ΣDBP	31.37	(31.26,	31.48)	33.09	(32.97,	33.21)	0.382

Abbreviations: MEHP (Mono-ethylhexyl phthalate); MEOHP (Mono-2-ethyl-5-oxohexyl phthalate); MEHHP (Mono-2-ethyl-5-hydroxylhexyl phthalate); MECPP (methylerythritol cyclodiphosphate); MCMHP (Mono(2-carboxymethylhexyl) phthalate); MBzP (monobenzyl phthalate);MEP (monoethyl phthalate); MiBP (monoisobutyl phthalate); MnBP (mono-n-butyl phthalate); MMP (Mono-methyl phthalate); MiNP (Mono-isooctyl phthalate); ΣHMW (high molecular weight) = (Mono-ethylhexyl phthalate (MEHP) + Mono-2-ethyl-5-oxohexyl phthalate (MEOHP) + Mono-2-ethyl-5-hydroxylhexyl phthalate (MEHHP) + Mono-benzyl phthalate (MBzP) + Mono-isooctyl phthalate (MiNP). ΣLMW (low molecular weight) = Mono-ethyl phthalate (MEP) + Mono-isobutyl phthalate (MiBP) + Mono-n-butyl phthalate (MnBP) + Mono-methyl phthalate (MMP). ∑DEHP (di(2-ethylhexyl) phthalate [molar sum of mono-(2-ethylhexyl) phthalate (MEHP), mono-(2-ethyl-5oxohexyl) phthalate (MEOHP), and mono-(2-ethyl-5-hydroxyhexyl) phthalate (MEHHP), and mono-(2-ethyl-5-carboxypentyl) phthalate (MECPP), and Mono(2-carboxymethylhexyl) phthalate (MCMHP]). ΣDBP (dibutyl phthalate) = Mono-isobutyl phthalate (MiBP) + Mono-n-butyl phthalate (MnBP).

**Table 3:** Prevalence odds ratios and 95% confidence intervals for metabolic syndromes in males and females based on urinary phthalate metabolites.

Phthalate Metabolites		Overall						Males						Females					
		(*n* = 1337)						(*n* = 693)						(*n* = 644)					
		Model 1 ^a^			Model 2 ^b^			Model 1 ^a^			Model 2 ^c^			Model 1 ^a^			Model 2 ^c^		
MEHP																			
	Q1	Ref			Ref			Ref			Ref			Ref			Ref		
	Q2	0.81	(0.52,	1.25)	0.63	(0.36,	1.12)	0.72	(0.44,	1.20)	0.66	(0.36,	1.20)	1.10	(0.42,	2.90)	1.43	(0.13,	15.46)
	Q3	1.10	(0.73,	1.65)	0.80	(0.44,	1.48)	0.93	(0.49,	1.73)	0.85	(0.41,	1.76)	0.99	(0.43,	2.30)	2.14	(0.25,	18.49)
	Q4	1.29	(0.86,	1.94)	0.72	(0.36,	1.43)	0.00	(0.00,	-----)	0.00	(0.00,	-----)	1.10	(0.49,	2.50)	2.01	(0.24,	17.04)
MEOHP																			
	Q1	Ref			Ref			Ref			Ref			Ref			Ref		
	Q2	0.81	(0.53,	1.23)	0.80	(0.49,	1.29)	0.83	(0.46,	1.47)	0.93	(0.48,	1.79)	0.75	(0.40,	1.38)	0.68	(0.33,	1.39)
	Q3	1.01	(0.67,	1.51)	0.88	(0.54,	1.42)	0.54	(0.28,	1.04) ^#^	0.48	(0.21,	1.09) *	1.31	(0.75,	2.29)	1.12	(0.59,	2.13)
	Q4	0.95	(0.63,	1.42)	0.64	(0.38,	1.07) *	0.96	(0.53,	1.75)	0.87	(0.41,	1.83)	0.86	(0.48,	1.52)	0.54	(0.26,	1.09) ^#^
MEHHP																			
	Q1	Ref			Ref			Ref			Ref			Ref			Ref		
	Q2	1.02	(0.68,	1.53)	1.16	(0.73,	1.84)	0.95	(0.54,	1.67)	1.19	(0.62,	2.30)	1.10	(0.61,	1.96)	1.12	(0.58,	2.17)
	Q3	0.85	(0.56,	1.30)	0.72	(0.43,	1.18)	0.57	(0.29,	1.12)	0.57	(0.25,	1.31)	1.01	(0.58,	1.77)	0.83	(0.43,	1.58)
	Q4	1.02	(0.68,	1.53)	0.83	(0.51,	1.36)	0.88	(0.48,	1.62)	0.81	(0.38,	1.71)	1.11	(0.64,	1.94)	0.87	(0.45,	1.68)
MECPP																			
	Q1	Ref			Ref			Ref			Ref			Ref			Ref		
	Q2	0.85	(0.56,	1.29)	0.79	(0.48,	1.31)	0.66	(0.37,	1.17)	0.65	(0.33,	1.28)	1.07	(0.57,	2.01)	1.22	(0.56,	2.70)
	Q3	1.07	(0.71,	1.59)	0.91	(0.56,	1.49)	0.82	(0.46,	1.47)	0.65	(0.32,	1.34)	1.23	(0.67,	2.24)	1.50	(0.70,	3.18)
	Q4	0.90	(0.59,	1.35)	0.74	(0.45,	1.23)	0.50	(0.25,	0.99) *	0.46	(0.20,	1.02) ^#^	1.16	(0.64,	2.10)	1.26	(0.59,	2.70)
MCMHP																			
	Q1	Ref			Ref			Ref			Ref			Ref			Ref		
	Q2	1.21	(0.79,	1.85)	1.22	(0.71,	2.09)	1.02	(0.56,	1.86)	1.21	(0.61,	2.37)	1.03	(0.50,	2.19)	1.73	(0.53,	5.71)
	Q3	1.38	(0.91,	2.10)	1.42	(0.83,	2.45)	1.15	0.62,	2.12)	1.22	(0.58,	2.56)	1.13	(0.56,	2.31)	2.13	(0.66,	6.95)
	Q4	1.35	(0.89,	2.05)	1.38	(0.79,	2.44)	1.06	(0.59,	1.92)	1.35	(0.62,	2.92)	1.23	(0.60,	2.53)	1.97	(0.59,	6.56)
MBzP																			
	Q1	Ref			Ref			Ref			Ref			Ref			Ref		
	Q2	0.59	(0.39,	0.91) *	0.65	(0.40,	1.04) ^#^	0.70	(0.37,	1.33)	0.85	(0.42,	1.73)	0.49	(0.27,	0.87) *	0.50	(0.26,	0.96) *
	Q3	0.93	(0.63,	1.39)	1.07	(0.66,	1.72)	0.97	(0.53,	1.76)	1.26	(0.61,	2.60)	0.84	(0.49,	1.44)	0.85	(0.45,	1.62)
	Q4	0.89	(0.60,	1.33)	0.88	(0.53,	1.46)	0.98	(0.54,	1.79)	1.55	(0.73,	3.29)	0.77	(0.45,	1.32)	0.55	(0.27,	1.09)
MEP																			
	Q1	Ref			Ref			Ref			Ref			Ref			Ref		
	Q2	1.51	(0.98,	2.32) ^#^	1.40	(0.81,	2.42)	2.29	(1.21,	4.33) ^*^	2.03	(0.93,	4.42) ^#^	0.99	(0.55,	1.80)	0.93	(0.43,	2.00)
	Q3	1.58	(1.03,	2.43) *	1.65	(0.98,	2.79) ^#^	1.75	(0.91,	3.39)	2.13	(1.01,	4.50) *	1.37	(0.78,	2.42)	1.22	(0.58,	2.57)
	Q4	1.45	(0.94,	2.24) ^#^	1.46	(0.86,	2.47)	1.52	(0.76,	3.01)	1.33	(0.59,	2.99)	1.27	(0.72,	2.24)	1.38	(0.67,	2.85)
MiBP																			
	Q1	Ref			Ref			Ref			Ref			Ref			Ref		
	Q2	0.71	(0.47,	1.07)	0.60	(0.37,	0.96) *	0.68	(0.37,	1.27)	0.61	(0.30,	1.23)	0.70	(0.40,	1.21)	0.59	(0.31,	1.13)
	Q3	0.93	(0.63,	1.37)	0.75	(0.47,	1.20)	0.75	(0.41,	1.36)	0.65	(0.31,	1.36)	1.05	(0.62,	1.79)	0.80	(0.43,	1.49)
	Q4	0.69	(0.46,	1.05) ^#^	0.64	(0.39,	1.06) ^#^	0.63	(0.34,	1.16)	0.68	(0.33,	1.43)	0.75	(0.42,	1.32)	0.61	(0.31,	1.23)
MnBP																			
	Q1	Ref			Ref			Ref			Ref			Ref			Ref		
	Q2	1.27	(0.84,	1.92)	1.26	(0.79,	2.00)	1.43	(0.76	,2.68)	1.46	(0.72,	2.96)	1.09	(0.63,	1.89)	1.11	(0.60,	2.08)
	Q3	1.38	(0.92,	2.07)	1.16	(0.71,	1.91)	1.74	(0.94,	3.22) ^#^	1.55	(0.74,	3.28)	1.11	(0.64,	1.92)	0.92	(0.47,	1.79)
	Q4	0.93	(0.60,	1.44)	0.89	(0.53,	1.49)	0.94	(0.48,	1.84)	1.02	(0.48,	2.18)	0.90	(0.50,	1.60)	0.81	(0.40,	1.65)
MMP																			
	Q1	Ref			Ref			Ref			Ref			Ref			Ref		
	Q2	1.12	(0.74,	1.71)	1.05	(0.61,	1.79)	1.19	(0.66,	2.15)	1.47	(0.74,	2.95)	0.63	(0.32,	1.24)	0.52	(0.22,	1.27)
	Q3	1.17	(0.77,	1.79)	1.06	(0.62,	1.82)	1.33	(0.74,	2.39)	1.33	(0.65,	2.72)	0.60	(0.30,	1.18)	0.57	(0.23,	1.37)
	Q4	1.30	(0.85,	1.98)	1.16	(0.67,	2.03)	1.09	(0.57,	2.08)	1.34	(0.61,	2.94)	0.76	(0.39,	1.49)	0.62	(0.25,	1.52)
MiNP																			
	Q1	Ref			Ref			Ref			Ref			Ref			Ref		
	Q2	0.98	(0.51,	1.86)	1.36	(0.63,	2.93)	0.41	(0.06,	2.63)	0.39	(0.06,	2.59)	1.13	(0.56,	2.26)	1.89	(0.79,	4.49)
	Q3	1.35	(0.59,	3.10)	1.81	(0.66,	4.95)	0.29	(0.02,	3.79)	0.28	(0.02,	3.77)	1.69	(0.69,	4.14)	2.76	(0.89,	8.52) ^#^
	Q4	1.43	(0.47,	4.31)	2.06	(0.55,	7.72)	0.76	(0.03,	19.12)	0.72	(0.03,	19.65)	1.58	(0.48,	5.18)	2.83	(0.65,	12.31)
ΣHMW																			
	Q1	Ref			Ref			Ref			Ref			Ref			Ref		
	Q2	0.87	(0.57,	1.33)	0.8	(0.47,	1.36)	0.61	(0.35,	1.08) ^#^	0.65	(0.33,	1.27)	1.13	(0.52,	2.46)	1.60	(0.49,	5.30)
	Q3	1.05	(0.70,	1.58)	0.81	(0.47,	1.39)	0.79	(0.43,	1.44)	0.65	(0.30,	1.40)	1.08	(0.51,	2.27)	1.48	(0.47,	4.72)
	Q4	1.16	(0.78,	1.75)	0.75	(0.43,	1.32)	0.82	(0.40,	1.67)	0.81	(0.35,	1.90)	1.10	(0.53,	2.28)	1.26	(0.40,	4.00)
ΣLMW																			
	Q1	Ref			Ref			Ref			Ref			Ref			Ref		
	Q2	1.97	(1.30,	2.98) **	1.65	(1.02,	2.66) *	2.03	(1.12,	3.66) *	1.96	(0.98,	3.88) ^#^	1.87	(1.04,	3.36) *	1.45	(0.74,	2.87)
	Q3	1.28	(0.83,	1.99)	1.13	(0.68,	1.89)	0.91	(0.46,	1.77)	0.88	(0.39,	1.97)	1.59	(0.87,	2.90)	1.31	(0.65,	2.63)
	Q4	1.25	(0.81,	1.95)	1.09	(0.65,	1.80)	1.01	(0.53,	1.93)	0.97	(0.46,	2.03)	1.47	(0.79,	2.71)	1.19	(0.58,	2.42)
ΣDEHP																			
	Q1	Ref			Ref			Ref			Ref			Ref			Ref		
	Q2	0.73	(0.47,	1.12)	0.62	(0.36,	1.06) ^#^	0.52	(0.29,	0.94) *	0.48	(0.23,	0.98) *	1.02	(0.48,	2.16)	1.41	(0.48,	4.16)
	Q3	1.23	(0.83,	1.83)	0.90	(0.54,	1.49)	0.82	(0.46,	1.46)	0.75	(0.38,	1.47)	1.59	(0.79,	3.22)	1.88	(0.66,	5.33)
	Q4	1.05	(0.70,	1.58)	0.69	(0.40,	1.18)	0.64	(0.31,	1.30)	0.52	(0.22,	1.24)	1.24	(0.62,	2.49)	1.41	(0.50,	3.99)
ΣDBP																			
	Q1	Ref			Ref			Ref			Ref			Ref			Ref		
	Q2	1.15	(0.76,	1.73)	1.09	(0.68,	1.74)	1.36	(0.73,	2.51)	1.40	(0.70,	2.81)	0.95	(0.54,	1.64)	0.88	(0.47,	1.64)
	Q3	1.26	(0.85,	1.89)	1.03	(0.64,	1.66)	1.43	(0.78,	2.65)	1.31	(0.64,	2.68)	1.10	(0.64,	1.90)	0.84	(0.44,	1.60)
	Q4	0.87	(0.56,	1.33)	0.85	(0.51,	1.40)	0.80	(0.42,	1.55)	0.90	(0.42,	1.90)	0.92	(0.52,	1.64)	0.80	(0.40,	1.60)

Abbreviations: MEHP (Mono-ethylhexyl phthalate); MEOHP (Mono-2-ethyl-5-oxohexyl phthalate); MEHHP (Mono-2-ethyl-5-hydroxylhexyl phthalate); MECPP (methylerythritol cyclodiphosphate); MCMHP (Mono(2-carboxymethylhexyl) phthalate); MBzP (monobenzyl phthalate);MEP (monoethyl phthalate); MiBP (monoisobutyl phthalate); MnBP (mono-n-butyl phthalate); MMP (Mono-methyl phthalate); MiNP (Mono-isooctyl phthalate); ΣHMW (high molecular weight) = (Mono-ethylhexyl phthalate (MEHP) + Mono-2-ethyl-5-oxohexyl phthalate (MEOHP) + Mono-2-ethyl-5-hydroxylhexyl phthalate (MEHHP) + Mono-benzyl phthalate (MBzP) + Mono-isooctyl phthalate (MiNP). ΣLMW (low molecular weight) = Mono-ethyl phthalate (MEP) + Mono-isobutyl phthalate (MiBP) + Mono-n-butyl phthalate (MnBP) + Mono-methyl phthalate (MMP). ∑DEHP (di(2-ethylhexyl) phthalate [molar sum of mono-(2-ethylhexyl) phthalate (MEHP), mono-(2-ethyl-5oxohexyl) phthalate (MEOHP), and mono-(2-ethyl-5-hydroxyhexyl) phthalate (MEHHP), and mono-(2-ethyl-5-carboxypentyl) phthalate (MECPP), and Mono(2-carboxymethylhexyl) phthalate (MCMHP]). ΣDBP (dibutyl phthalate) = Mono-isobutyl phthalate (MiBP) + Mono-n-butyl phthalate (MnBP). ^#^ *p* < 0.10 * *p* < 0.05 ** *p* < 0.01. ^a^ Adjusted for urinary creatinine. ^b^ Adjusted for urinary creatinine, age, sex, education, and working status. ^c^ Adjusted for the same variables in b, except sex.

**Table 4:** Prevalence odds ratios and 95% confidence intervals for individual MetS components for each quartile of urinary phthalate metabolite concentrations.

		High Blood Pressure			Low HDL Cholesterol			High Waist Circumference			Hyperglycemia			Elevated Triglycerides		
*n* with/without component		308/1029			381/956			601/736			267/1070			313/1024		
MEHP	Q1	Ref			Ref			Ref			Ref			Ref		
	Q2	0.71	(0.45,	1.12)	1.06	(0.59,	1.90)	0.76	(0.50,	1.14)	0.54	(0.33,	0.88) *	0.78	(0.49,	1.24)
	Q3	0.85	(0.50,	1.43)	1.17	(0.64,	2.17)	0.84	(0.53,	1.33)	0.55	(0.31,	0.95) *	0.89	(0.53,	1.47)
	Q4	0.83	(0.44,	1.58)	1.07	(0.56,	2.07)	0.62	(0.36,	1.04) ^#^	0.88	(0.47,	1.64)	0.76	(0.42,	1.38)
MEOHP	Q1	Ref			Ref			Ref			Ref			Ref		
	Q2	0.92	(0.59,	1.44)	0.88	(0.57,	1.36)	0.92	(0.64,	1.33)	0.76	(0.49,	1.20)	0.90	(0.60,	1.36)
	Q3	1.24	(0.79.	1.95)	0.68	(0.44,	1.06) ^#^	0.93	(0.64,	1.35)	0.88	(0.56,	1.38)	0.81	(0.53,	1.24)
	Q4	0.73	(0.45,	1.19)	0.71	(0.45,	1.10)	0.85	(0.58,	1.25)	0.97	(0.62,	1.53)	0.82	(0.53,	1.27)
MEHHP	Q1	Ref			Ref			Ref			Ref			Ref		
	Q2	1.00	(0.64,	1.56)	1.05	(0.69,	1.61)	0.94	(0.65,	1.35)	0.75	(0.48,	1.16)	1.36	(0.91,	2.03)
	Q3	0.97	(0.61,	1.54)	0.87	(0.57,	1.32)	0.86	(0.60,	1.24)	0.62	(0.39,	0.99) *	0.82	(0.53,	1.26)
	Q4	0.95	(0.60,	1.50)	0.84	(0.55,	1.30)	0.93	(0.64,	1.34)	0.98	(0.63,	1.50)	0.87	(0.56,	1.34)
MECPP	Q1	Ref			Ref			Ref			Ref			Ref		
	Q2	0.96	(0.61,	1.50)	1.44	(0.92,	2.26)	0.75	(0.51,	1.09)	0.69	(0.43,	1.10)	1.27	(0.83,	1.94)
	Q3	1.05	(0.66,	1.66)	0.93	(0.59,	1.47)	1.15	(0.79,	1.67)	0.78	(0.49,	1.24)	1.07	(0.69,	1.67)
	Q4	0.74	(0.46,	1.19)	0.98	(0.62,	1.55)	0.83	(0.57,	1.21)	0.96	(0.61,	1.49)	1.15	(0.74,	1.78)
MCMHP	Q1	Ref			Ref			Ref			Ref			Ref		
	Q2	1.05	(0.67,	1.65)	0.99	(0.61,	1.63)	1.12	(0.76,	1.65)	1.45	(0.90,	2.34)	0.92	(0.59,	1.44)
	Q3	1.04	(0.65,	1.68)	0.91	(0.55,	1.51)	1.39	(0.93,	2.08)	1.42	(0.86,	2.34)	1.21	(0.77,	1.90)
	Q4	0.88	(0.53,	1.47)	1.12	(0.67,	1.87)	1.15	(0.76,	1.74)	1.72	(1.04,	2.84) *	1.14	(0.71,	1.82)
MBzP	Q1	Ref			Ref			Ref			Ref			Ref		
	Q2	0.80	(0.52,	1.23)	0.73	(0.49,	1.10)	1.04	(0.74,	1.47)	0.56	(0.37,	0.86) **	0.74	(0.50,	1.09)
	Q3	0.94	(0.59,	1.49)	0.68	(0.44,	1.05) ^#^	1.03	(0.71,	1.49)	0.86	(0.56,	1.33)	0.73	(0.48,	1.12)
	Q4	0.96	(0.59,	1.55)	0.65	(0.41,	1.01) ^#^	1.09	(0.74,	1.60)	0.60	(0.37,	0.97) *	0.59	(0.37,	0.92) *
MEP	Q1	Ref			Ref			Ref			Ref			Ref		
	Q2	0.94	(0.58,	1.52)	0.96	(0.60,	1.53)	0.92	(0.62,	1.35)	1.74	(1.06,	2.85) *	1.20	(0.77,	1.86)
	Q3	1.11	(0.70,	1.75)	1.09	(0.69,	1.72)	1.19	(0.81,	1.73)	1.35	(0.82,	2.22)	1.07	(0.69,	1.65)
	Q4	0.97	(0.61,	1.54)	1.47	(0.94,	2.30) ^#^	0.93	(0.64,	1.35)	1.73	(1.07,	2.80) *	1.08	(0.70,	1.68)
MiBP	Q1	Ref			Ref			Ref			Ref			Ref		
	Q2	0.68	(0.44,	1.06) ^#^	0.75	(0.49,	1.14)	0.82	(0.58,	1.16)	0.66	(0.42,	1.03) ^#^	0.92	(0.61,	1.38)
	Q3	0.87	(0.56,	1.36)	0.96	(0.63,	1.46)	0.96	(0.67,	1.38)	1.00	(0.65,	1.54)	1.05	(0.69,	1.59)
	Q4	0.85	(0.54,	1.36)	0.89	(0.57,	1.38)	0.57	(0.39,	0.84) **	1.00	(0.64,	1.56)	1.01	(0.65,	1.55)
MnBP	Q1	Ref			Ref			Ref			Ref			Ref		
	Q2	1.57	(1.01,	2.42) *	1.11	(0.73,	1.69)	0.93	(0.65,	1.33)	1.13	(0.73,	1.74)	1.25	(0.83,	1.90)
	Q3	1.05	(0.65,	1.71)	1.21	(0.78,	1.86)	0.79	(0.54,	1.14)	1.04	(0.66,	1.64)	1.54	(1.01,	2.36) *
	Q4	1.10	(0.69,	1.75)	1.13	(0.73,	1.75)	0.78	(0.54,	1.13)	0.96	(0.61,	1.52)	1.07	(0.69,	1.66)
MMP	Q1	Ref			Ref			Ref			Ref			Ref		
	Q2	0.85	(0.53	1.36)	1.01	(0.62,	1.67)	0.93	(0.63,	1.38)	1.12	(0.69,	1.83)	0.89	(0.57,	1.39)
	Q3	0.92	(0.57,	1.49)	1.17	(0.71,	1.93)	1.00	(0.68,	1.48)	1.43	(0.88,	2.32)	1.00	(0.65,	1.56)
	Q4	1.25	(0.77,	2.04)	1.02	(0.61,	1.73)	0.98	(0.65,	1.49)	1.42	(0.85,	2.37)	0.75	(0.46,	1.21)
MiNP	Q1	Ref			Ref			Ref			Ref			Ref		
	Q2	0.76	(0.35,	1.65)	1.00	(0.54,	1.85)	0.98	(0.55,	1.77)	0.67	(0.32,	1.40)	1.74	(0.85,	3.56)
	Q3	0.91	(0.33,	2.54)	1.22	(0.53,	2.81)	0.98	(0.45,	2.16)	0.77	(0.29,	2.06)	3.68	(1.45,	9.35) **
	Q4	0.97	(0.25,	3.77)	1.44	(0.47,	4.35)	1.10	(0.38,	3.17)	0.71	(0.19,	2.66)	2.80	(0.81,	9.70)
ΣHMW	Q1	Ref			Ref			Ref			Ref			Ref		
	Q2	1.14	(0.72,	1.80)	0.98	(0.59,	1.63)	0.93	(0.63,	1.38)	0.54	(0.33,	0.88) *	1.00	(0.65,	1.55)
	Q3	1.16	(0.71,	1.87)	0.72	(0.43,	1.22)	1.01	(0.67,	1.52)	0.55	(0.33,	0.91) *	0.76	(0.48,	1.21)
	Q4	0.81	(0.48,	1.38)	0.72	(0.43,	1.23)	0.85	(0.56,	1.30)	0.92	(0.56,	1.50)	0.78	(0.48,	1.27)
ΣLMW	Q1	Ref			Ref			Ref			Ref			Ref		
	Q2	1.22	(0.78,	1.92)	1.37	(0.88,	2.11)	0.85	(0.59,	1.22)	1.23	(0.79,	1.92)	1.24	(0.82,	1.89)
	Q3	1.11	(0.70,	1.77)	1.28	(0.83,	2.00)	0.94	(0.65,	1.36)	0.94	(0.59,	1.50)	1.06	(0.69,	1.63)
	Q4	0.98	(0.62,	1.55)	1.48	(0.96,	2.29) ^#^	0.71	(0.50,	1.03) ^#^	1.19	(0.76,	1.85)	1.03	(0.68,	1.58)
ΣDEHP	Q1	Ref			Ref			Ref			Ref			Ref		
	Q2	0.90	(0.57,	1.43)	0.82	(0.50,	1.34)	0.90	(0.61,	1.33)	0.81	(0.51,	1.30)	1.11	(0.72,	1.72)
	Q3	1.25	(0.79,	1.98)	0.74	(0.45,	1.21)	1.06	(0.72,	1.57)	0.71	(0.43,	1.15)	0.96	(0.61,	1.50)
	Q4	0.69	(0.41,	1.15)	0.66	(0.40,	1.09)	0.86	(0.57,	1.30)	1.11	(0.69,	1.80)	0.88	(0.55,	1.42)
ΣDBP	Q1	Ref			Ref			Ref			Ref			Ref		
	Q2	0.94	(0.60,	1.47)	1.21	(0.80,	1.84)	1.01	(0.71,	1.43)	1.05	(0.68,	1.62)	1.32	(0.87,	1.99)
	Q3	1.14	(0.73,	1.78)	1.29	(0.84,	1.98)	0.74	(0.51,	1.06)	1.01	(0.65,	1.58)	1.28	(0.84,	1.95)
	Q4	0.85	(0.53,	1.35)	1.28	(0.82,	1.99)	0.77	(0.53,	1.12)	0.89	(0.56,	1.41)	1.27	(0.82,	1.95)

Abbreviations: MEHP (Mono-ethylhexyl phthalate); MEOHP (Mono-2-ethyl-5-oxohexyl phthalate); MEHHP (Mono-2-ethyl-5-hydroxylhexyl phthalate); MECPP (methylerythritol cyclodiphosphate); MCMHP (Mono(2-carboxymethylhexyl) phthalate); MBzP (monobenzyl phthalate);MEP (monoethyl phthalate); MiBP (monoisobutyl phthalate); MnBP (mono-n-butyl phthalate); MMP (Mono-methyl phthalate); MiNP (Mono-isooctyl phthalate); ΣHMW (high molecular weight) = (Mono-ethylhexyl phthalate (MEHP) + Mono-2-ethyl-5-oxohexyl phthalate (MEOHP) + Mono-2-ethyl-5-hydroxylhexyl phthalate (MEHHP) + Mono-benzyl phthalate (MBzP) + Mono-isooctyl phthalate (MiNP). ΣLMW (low molecular weight) = Mono-ethyl phthalate (MEP) + Mono-isobutyl phthalate (MiBP) + Mono-n-butyl phthalate (MnBP) + Mono-methyl phthalate (MMP). ∑DEHP (di(2-ethylhexyl) phthalate [molar sum of mono-(2-ethylhexyl) phthalate (MEHP), mono-(2-ethyl-5oxohexyl) phthalate (MEOHP), and mono-(2-ethyl-5-hydroxyhexyl) phthalate (MEHHP), and mono-(2-ethyl-5-carboxypentyl) phthalate (MECPP), and Mono(2-carboxymethylhexyl) phthalate (MCMHP]). ΣDBP (dibutyl phthalate) = Mono-isobutyl phthalate (MiBP) + Mono-n-butyl phthalate (MnBP). ^#^ *p* < 0.10 * *p* < 0.05 ** *p* < 0.01. Adjusted for urinary creatinine, age, sex, education, and working status.

**Table 5:** Prevalence odds ratios and 95% confidence intervals for MetS stratified by age and sex.

Phthalate Metabolites		Males						Females					
		<50 Years			≥50 Years			<50 Years			≥50 Years		
		(*n* = 336)			(*n* = 357)			(*n* = 333)			(*n* = 311)		
MEOHP	Q1	Ref			Ref			Ref			Ref		
	Q2	0.94	(0.31,	2.84)	0.98	(0.42,	2.24)	0.32	(0.10,	0.97) *	1.11	(0.40,	3.08)
	Q3	0.43	(0.10,	1.80)	0.60	(0.21,	1.68)	0.76	(0.30,	1.95)	1.43	(0.56,	3.67)
	Q4	0.78	(0.21,	2.95)	1.02	(0.40,	2.59)	0.31	(0.10,	0.93) *	0.79	(0.29,	2.16)
MBzP	Q1	Ref			Ref			Ref			Ref		
	Q2	0.85	(0.25,	2.86)	0.75	(0.30,	1.87)	0.04	(0.01,	0.31) *	1.42	(0.59,	3.44)
	Q3	2.17	(0.69,	6.85)	0.82	(0.30,	2.22)	0.37	(0.15,	0.93) *	1.78	(0.69,	4.592)
	Q4	1.18	(0.26,	5.28)	1.73	(0.71,	4.24)	0.31	(0.12,	0.82) *	0.98	(0.35,	2.75)
MEP	Q1	Ref			Ref			Ref			Ref		
	Q2	0.84	(0.23,	3.13)	3.11	(1.12,	8.63) *	1.76	(0.48,	6.39)	0.55	(0.20,	1.54)
	Q3	1.95	(0.62,	6.15)	2.32	(0.84,	6.37)	1.75	(0.49,	6.33)	0.98	(0.38,	2.58)
	Q4	0.54	(0.12,	2.39)	2.07	(0.74,	5.79)	1.68	(0.49,	5.83)	1.16	(0.45,	2.99)
MiBP	Q1	Ref			Ref			Ref			Ref		
	Q2	0.18	(0.05,	0.75) *	1.02	(0.43,	2.43)	0.47	(0.17,	1.31)	0.58	(0.24,	1.42)
	Q3	0.37	(0.11,	1.26)	0.96	(0.37,	2.50)	1.00	(0.38,	2.61)	0.58	(0.24,	1.36)
	Q4	0.67	(0.19,	2.38)	0.90	(0.35,	2.29)	0.32	(0.10,	1.05) ^#^	0.73	(0.29,	1.83)
ΣDEHP	Q1	Ref			Ref			Ref			Ref		
	Q2	0.71	(0.24,	2.11)	0.35	(0.12,	0.98) *	0.87	(0.19,	3.91)	1.85	(0.35,	9.87)
	Q3	1.08	(0.33,	3.47)	0.77	(0.32,	1.85)	0.66	(0.15,	3.00)	3.97	(0.82,	19.36) ^#^
	Q4	0.24	(0.03,	1.96)	0.80	(0.29,	2.18)	0.60	(0.13,	2.68)	2.66	(0.54,	13.05)

Abbreviations: MEOHP (Mono-2-ethyl-5-oxohexyl phthalate); MBzP (monobenzyl phthalate); MEP (monoethyl phthalate); MiBP (monoisobutyl phthalate); ∑DEHP (di(2-ethylhexyl) phthalate [molar sum of mono-(2-ethylhexyl) phthalate (MEHP), mono-(2-ethyl-5oxohexyl) phthalate (MEOHP), and mono-(2-ethyl-5-hydroxyhexyl) phthalate (MEHHP), and mono-(2-ethyl-5-carboxypentyl) phthalate (MECPP), and Mono(2-carboxymethylhexyl) phthalate (MCMHP]). ^#^ *p* < 0.10 * *p* < 0.05. Adjusted for urinary creatinine, age, education, and working status.

## Data Availability

Data supporting the findings of this study are available from the Taiwan Biobank, but restrictions apply to the availability of these data, which were used under the license of the current study and are, therefore, not public. However, data are available from the corresponding authors upon reasonable request and with permission from the Taiwan Biobank.

## Supplementary Materials

The following supporting information can be downloaded at: https://www.mdpi.com/article/10.3390/ijerph191610458/s1. Table S1: Prevalence odds ratios and 95% confidence intervals for individual MetS components for each quartile of urinary phthalate metabolite; Table S2: Prevalence odds ratios for the association between urinary metabolites concentrations and metabolic syndrome stratified by menopausal status in females.
