# Supplementary material for: Sex Differences between Urinary Phthalate Metabolites and Metabolic Syndrome in Adults: A Cross-Sectional Taiwan Biobank Study
Source: Int J Environ Res Public Health. 2022 Aug 22;19(16):10458. doi: 10.3390/ijerph191610458 (PMC9407747; doi:10.3390/ijerph191610458)
Supplement: Supplementary file 1 [file ijerph-19-10458-s001.zip › ijerph-1809394-supplementary.pdf]

Supplemental Table S1 Prevalence odds ratios and 95 % confidence intervals for individual MetS components for each quartile of urinary phthalate metabolite.

		high Blood Pressure			Low HDL Cholesterol			High Waist Circumference			Hyperglycemia			Elevated Triglycerides		
		Overall	Males	Females	Overall	Males	Females	Overall	Males	Females	Overall	Males	Females	Overall	Males	Females
	n with component/ n without component	308/1029	212/481	96/548	381/956	88/605	293/351	601/736	271/422	314/330	267/1070	140/553	127/517	313/1024	159/534	154/490
MEHP	Q1	Ref	Ref	Ref	Ref	Ref	Ref	Ref	Ref	Ref	Ref	Ref	Ref	Ref	Ref	Ref
	Q2	0.71 (0.45 , 1.12)	0.72 (0.45 , 1.16)	0.34 (0.04 , 2.78)	1.06 (0.59 , 1.90)	0.82 (0.43 , 1.56)	3.20 (0.53 , 19.23)	0.76 (0.50 , 1.14)	0.72 (0.46 , 1.11)	0.10 (0.74 , 4.56)	0.54 (0.33 , 0.88)*	0.54 (0.32 , 0.91)*	1.41 (0.13 , 14.98)	0.78 (0.49 , 1.24)	0.78 (0.48 , 1.28)	0.98 (0.15 , 6.35)
	Q3	0.85 (0.50 , 1.43)	0.99 (0.56 , 1.77)	0.43 (0.07 , 2.46)	1.17 (0.64 , 2.17)	1.20 (0.57 , 2.50)	2.64 (0.51 , 13.73)	0.84 (0.53 , 1.33)	0.79 (0.46 , 1.34)	0.07 (0.90 , 4.74)#	0.55 (0.31 , 0.95)*	0.58 (0.30 , 1.11)	1.35 (0.16 , 11.71)	0.89 (0.53 , 1.47)	0.97 (0.54 , 1.74)	1.08 (0.20 , 5.66)
	Q4	0.83 (0.44 , 1.58)	0.36 (0.10 , 1.35)	0.54 (0.10 , 3.02)	1.07 (0.56 , 2.07)	0.00 (0.00 , .)	2.73 (0.53 , 13.96)	0.62 (0.36 , 1.04)#	0.34 (0.11 , 1.07)#	0.15 (0.65 , 3.40)	0.88 (0.47 , 1.64)	0.49 (0.13 , 1.80)	2.22 (0.26 , 18.56)	0.76 (0.42 , 1.38)	0.32 (0.07 , 1.44)	1.00 (0.20 , 5.18)
MEOHP	Q1	Ref	Ref	Ref	Ref	Ref	Ref	Ref	Ref	Ref	Ref	Ref	Ref	Ref	Ref	Ref
	Q2	0.92 (0.59 , 1.44)	0.94 (0.55 , 1.61)	0.87 (0.37 , 2.06)	0.88 (0.57 , 1.36)	1.18 (0.59 , 2.36)	0.74 (0.42 , 1.30)	0.92 (0.64 , 1.33)	0.91 (0.56 , 1.50)	0.89 (0.50 , 1.58)	0.76 (0.49 , 1.20)	0.69 (0.39 , 1.23)	1.00 (0.46 , 2.15)	0.90 (0.59 , 1.36)	1.20 (0.70 , 2.06)	0.61 (0.32 , 1.18)
	Q3	1.24 (0.79 , 1.95)	1.24 (0.70 , 2.18)	1.26 (0.58 , 2.76)	0.68 (0.44 , 1.06)#	0.55 (0.23 , 1.30)	0.66 (0.38 , 1.14)	0.93 (0.64 , 1.35)	0.91 (0.54 , 1.54)	0.84 (0.48 , 1.47)	0.88 (0.56 , 1.38)	0.45 (0.23 , 0.90)*	1.68 (0.83 , 3.38)	0.81 (0.53 , 1.24)	0.83 (0.45 , 1.51)	0.71 (0.38 , 1.32)
	Q4	0.73 (0.45 , 1.19	0.67 (0.36 , 1.26)	0.80 (0.35 , 1.83)	0.71 (0.45 , 1.10)	0.88 (0.40 , 1.94)	0.60 (0.34 , 1.03)#	0.85 (0.58 , 1.25)	0.90 (0.52 , 1.56)	0.79 (0.45 , 1.38)	0.97 (0.62 , 1.53)	1.03 (0.56 , 1.89)	1.20 (0.58 , 2.50)	0.82 (0.53 , 1.27)	0.80 (0.43 , 1.52)	0.75 (0.41 , 1.39)
MEHHP	Q1	Ref	Ref	Ref	Ref	Ref	Ref	Ref	Ref	Ref	Ref	Ref	Ref	Ref	Ref	Ref
	Q2	1.00 (0.64 , 1.56)	1.25 (0.73 , 2.15)	0.62 (0.28 , 1.41)	1.05 (0.69 , 1.61)	1.22 (0.60 , 2.49)	0.99 (0.58 , 1.69)	0.94 (0.65 , 1.35)	1.25 (0.76 , 2.06)	0.63 (0.36 , 1.09)#	0.75 (0.48 , 1.16)	0.61 (0.34 , 1.09)	0.97 (0.49 , 1.93)	1.36 (0.91 , 2.03)	1.14 (0.66 , 1.96)	1.70 (0.93 , 3.12)#
	Q3	0.97 (0.61 , 1.54)	0.98 (0.54 , 1.80)	0.90 (0.43 , 1.85)	0.87 (0.57 , 1.32)	0.94 (0.42 , 2.09)	0.82 (0.49 , 1.35)	0.86 (0.60 , 1.24)	0.92 (0.53 , 1.59)	0.76 (0.46 , 1.27)	0.62 (0.39 , 0.99)*	0.48 (0.24 , 0.94)*	0.84 (0.43 , 1.62)	0.82 (0.53 , 1.26)	0.85 (0.46 , 1.58)	0.86 (0.47 , 1.59)
	Q4	0.95 (0.60 , 1.50)	1.04 (0.58 , 1.87)	0.77 (0.36 , 1.65)	0.84 (0.55 , 1.30)	0.78 (0.35 , 1.76)	0.83 (0.49 , 1.39)	0.93 (0.64 , 1.34)	1.08 (0.64 , 1.83)	0.75 (0.44 , 1.28)	0.98 (0.63 , 1.50)	0.77 (0.42 , 1.40)	1.42 (0.75 , 2.70)	0.87 (0.56 , 1.34)	0.81 (0.44 , 1.48)	0.94 (0.50 , 1.75)
MECPP	Q1	Ref	Ref	Ref	Ref	Ref	Ref	Ref	Ref	Ref	Ref	Ref	Ref	Ref	Ref	Ref
	Q2	0.96 (0.61 , 1.50)	0.77 (0.45 , 1.33)	1.57 (0.67 , 3.67)	1.44 (0.92 , 2.26)	1.52 (0.77 , 3.00)	1.47 (0.81 , 2.66)	0.75 (0.51 , 1.09)	0.75 (0.46 , 1.23)	0.80 (0.44 , 1.47)	0.69 (0.43 , 1.10)	0.50 (0.27 , 0.93)*	1.18 (0.54 , 2.57)	1.27 (0.83 , 1.94)	1.07 (0.62 , 1.84)	1.93 (0.91 , 4.11)#
	Q3	1.05 (0.66 , 1.66)	1.25 (0.72 , 2.19)	0.95 (0.40 , 2.26)	0.93 (0.59 , 1.47)	0.57 (0.24 , 1.37)	1.09 (0.61 , 1.95)	1.15 (0.79 , 1.67)	1.16 (0.69 , 1.94)	1.26 (0.70 , 2.26)	0.78 (0.49 , 1.24)	0.52 (0.27 , 0.99)*	1.34 (0.63 , 2.84)	1.07 (0.69 , 1.67)	0.90 (0.50 , 1.62)	1.61 (0.76 , 3.38)
	Q4	0.74 (0.46 , 1.19)	0.68 (0.37 , 1.24)	0.87 (0.37 , 2.05)	0.98 (0.62 , 1.55)	0.84 (0.38 , 1.87)	1.06 (0.59 , 1.87)	0.83 (0.57 , 1.21)	0.70 (0.41 , 1.19)	0.96 (0.54 , 1.71)	0.96 (0.61 , 1.49)	0.99 (0.55 , 1.78)	1.28 (0.60 , 2.71)	1.15 (0.74 , 1.78)	0.85 (0.47 , 1.56)	1.88 (0.91 , 3.92)#
MCMHP	Q1	Ref	Ref	Ref	Ref	Ref	Ref	Ref	Ref	Ref	Ref	Ref	Ref	Ref	Ref	Ref
	Q2	1.05 (0.67 , 1.65)	1.02 (0.60 , 1.74)	1.02 (0.33 , 3.14)	0.99 (0.61 , 1.63)	0.92 (0.45 , 1.86)	1.35 (0.59 , 3.10)	1.12 (0.76 , 1.65)	1.24 (0.77 , 2.01)	0.89 (0.39 , 2.06)	1.45 (0.90 , 2.34)	1.45 (0.82 , 2.55)	1.93 (0.54 , 6.89)	0.92 (0.59 , 1.44)	0.95 (0.54 , 1.65)	0.92 (0.35 , 2.40)
	Q3	1.04 (0.65 , 1.68)	0.89 (0.49 , 1.62)	1.08 (0.36 , 3.32)	0.91 (0.55 , 1.51)	0.65 (0.27 , 1.55)	1.32 (0.58 , 3.00)	1.39 (0.93 , 2.08)	1.71 (1.00 , 2.92)*	1.14 (0.50 , 2.61)	1.42 (0.86 , 2.34)	1.20 (0.63 , 2.29)	2.04 (0.57 , 7.24)	1.21 (0.77 , 1.90)	1.14 (0.63 , 2.08)	1.22 (0.48 , 3.15)
	Q4	0.88 (0.53 , 1.47)	1.28 (0.70 , 2.36)	0.53 (0.16 , 1.79)	1.12 (0.67 , 1.87)	0.99 (0.45 , 2.16)	1.57 (0.68 , 3.62)	1.15 (0.76 , 1.74)	0.96 (0.54 , 1.69)	1.05 (0.45 , 2.45)	1.72 (1.04 , 2.84)*	1.64 (0.85 , 3.13)	2.40 (0.67 , 8.60)	1.14 (0.71 , 1.82)	1.27 (0.69 , 2.34)	1.08 (0.41 , 2.85)
MBzP	Q1	Ref	Ref	Ref	Ref	Ref	Ref	Ref	Ref	Ref	Ref	Ref	Ref	Ref	Ref	Ref
	Q2	0.80 (0.52 , 1.23)	0.67 (0.40 , 1.14)	1.22 (0.56 , 2.63)	0.73 (0.49 , 1.10)	0.67 (0.33 , 1.36)	0.71 (0.43 , 1.19)	1.04 (0.74 , 1.47)	1.12 (0.69 , 1.82)	0.96 (0.57 , 1.62)	0.56 (0.36 , 0.86)	0.61 (0.34 , 1.10)#	0.50 (0.26 , 0.96)*	0.74 (0.50 , 1.09)	0.74 (0.44 , 1.26)	0.72 (0.40 , 1.29)
	Q3	0.94 (0.59 , 1.49)	0.79 (0.44 , 1.41)	1.31 (0.59 , 2.93)	0.68 (0.44 , 1.05)#	0.67 (0.31 , 1.46)	0.65 (0.38 , 1.11)	1.03 (0.71 , 1.49)	1.32 (0.78 , 2.22)	0.77 (0.45 , 1.34)	0.86 (0.56 , 1.33)	0.73 (0.39 , 1.37)	0.96 (0.51 , 1.80)	0.73 (0.48 , 1.12)	0.65 (0.36 , 1.19)	0.79 (0.43 , 1.45)
	Q4	0.96 (0.59 , 1.55)	0.95 (0.52 , 1.76)	1.05 (0.46 , 2.42)	0.65 (0.41 , 1.01)#	0.71 (0.31 , 1.64)	0.60 (0.35 , 1.04)#	1.09 (0.74 , 1.60)	1.16 (0.66 , 2.06)	0.95 (0.55 , 1.64)	0.60 (0.37 , 0.97)*	0.88 (0.46 , 1.70)	0.43 (0.21 , 0.89)*	0.59 (0.37 , 0.92)*	0.71 (0.37 , 1.37)	0.50 (0.26 , 0.96)*
MEP	Q1	Ref	Ref	Ref	Ref	Ref	Ref	Ref	Ref	Ref	Ref	Ref	Ref	Ref	Ref	Ref
	Q2	0.94 (0.58 , 1.52)	0.78 (0.43 , 1.39)	1.74 (0.66 , 4.58)	0.96 (0.60 , 1.53)	1.35 (0.60 , 3.04)	0.82 (0.46 , 1.47)	0.92 (0.62 , 1.35)	0.86 (0.51 , 1.47)	1.00 (0.55 , 1.79)	1.74 (1.06 , 2.85)*	2.23 (1.19 , 4.16)*	1.40 (0.61 , 3.18)	1.20 (0.77 , 1.86)	2.04 (1.13 , 3.71)*	0.60 (0.31 , 1.17)
	Q3	1.11 (0.70 , 1.75)	0.84 (0.48 , 1.46)	2.34 (0.92 , 5.98)#	1.09 (0.69 , 1.72)	1.77 (0.83 , 3.78)	0.85 (0.48 , 1.52)	1.19 (0.81 , 1.73)	0.82 (0.49 , 1.37)	1.78 (0.98 , 3.21)#	1.35 (0.82 , 2.22)	1.22 (0.64 , 2.34)	1.55 (0.69 , 3.49)	1.07 (0.69 , 1.65)	1.46 (0.81 , 2.65)	0.69 (0.36 , 1.33)
	Q4	0.97 (0.61 , 1.54)	0.72 (0.41 , 1.28)	2.18 (0.85 , 5.56)	1.47 (0.94 , 2.30)#	1.17 (0.51 , 2.65)	1.64 (0.93 , 2.91)#	0.93 (0.64 , 1.35)	1.04 (0.62 , 1.73)	0.91 (0.51 , 1.61)	1.73 (1.07 , 2.80)*	1.34 (0.70 , 2.56)	2.14 (0.98 , 4.67)#	1.08 (0.70 , 1.68)	1.07 (0.57 , 1.98)	0.89 (0.48 , 1.68)
MiBP	Q1	Ref	Ref	Ref	Ref	Ref	Ref	Ref	Ref	Ref	Ref	Ref	Ref	Ref	Ref	Ref
	Q2	0.68 (0.44 , 1.06)#	0.62 (0.35 , 1.08)#	0.82 (0.39 , 1.73)	0.75 (0.49 , 1.14)	1.02 (0.48 , 2.15)	0.64 (0.38 , 1.07)#	0.82 (0.58 , 1.16)	0.78 (0.48 , 1.28)	0.89 (0.53 , 1.49)	0.66 (0.42 , 1.03)#	0.58 (0.31 , 1.10)	0.73 (0.38 , 1.42)	0.92 (0.61 , 1.38)	1.14 (0.66 , 1.98)	0.67 (0.37 , 1.23)
	Q3	0.87 (0.56 , 1.36)	0.75 (0.42 , 1.33)	1.08 (0.53 , 2.21)	0.96 (0.63 , 1.46)	1.26 (0.59 , 2.68)	0.82 (0.49 , 1.37)	0.96 (0.67 , 1.38)	0.75 (0.45 , 1.26)	1.18 (0.69 , 2.00)	1.00 (0.65 , 1.54)	1.22 (0.68 , 2.19)	0.87 (0.45 , 1.66)	1.05 (0.69 , 1.59)	1.33 (0.75 , 2.37)	0.76 (0.42 , 1.39)
	Q4	0.85 (0.54 , 1.36)	1.06 (0.60 , 1.87)	0.54 (0.23 , 1.26)	0.89 (0.57 , 1.38)	1.33 (0.62 , 2.89)	0.72 (0.42 , 1.24)	0.57 (0.39 , 0.84)*	0.54 (0.31 , 0.93)*	0.63 (0.36 , 1.10)	1.00 (0.64 , 1.56)	0.85 (0.45 , 1.60)	1.21 (0.62 , 2.34)	1.01 (0.66 , 1.55)	0.87 (0.47 , 1.62)	1.06 (0.57 , 1.96)

Supplemental Table 1 (continued)

		high Blood Pressure			Low HDL Cholesterol			High Waist Circumference			Hyperglycemia			Elevated Triglycerides		
		Overall	Males	Females	Overall	Males	Females	Overall	Males	Females	Overall	Males	Females	Overall	Males	Females
	n with component/ n without component	308/1029	212/481	96/548	381/956	88/605	293/351	601/736	271/422	314/330	267/1070	140/553	127/517	313/1024	159/534	154/490
MnBP	Q1	Ref	Ref	Ref	Ref	Ref	Ref	Ref	Ref	Ref	Ref	Ref	Ref	Ref	Ref	Ref
	Q2	1.57 (1.01 , 2.42)*	1.38 (0.80 , 2.38)	1.99 (0.93 , 4.27)#	1.11 (0.73 , 1.69)	1.53 (0.68 , 3.40)	0.95 (0.57 , 1.59)	0.93 (0.65 , 1.33)	1.00 (0.61 , 1.65)	0.85 (0.50 , 1.43)	1.13 (0.73 , 1.74)	1.04 (0.57 , 1.88)	1.26 (0.67 , 2.39)	1.25 (0.83 , 1.90)	1.26 (0.70 , 2.25)	1.26 (0.69 , 2.30)
	Q3	1.05 (0.65 , 1.71)	0.88 (0.47 , 1.63)	1.35 (0.59 , 3.08)	1.21 (0.78 , 1.86)	2.04 (0.91 , 4.53)#	0.93 (0.55 , 1.57)	0.79 (0.54 , 1.14)	0.85 (0.50 , 1.45)	0.72 (0.42 , 1.22)	1.04 (0.66 , 1.64)	1.25 (0.67 , 2.35)	0.97 (0.49 , 1.92)	1.54 (1.01 , 2.36)*	1.67 (0.92 , 3.03)#	1.36 (0.73 , 2.51)
	Q4	1.10 (0.69 , 1.75)	1.09 (0.63 , 1.91)	1.13 (0.47 , 2.71)	1.13 (0.73 , 1.75)	1.94 (0.90 , 4.21)#	0.86 (0.50 , 1.48)	0.78 (0.54 , 1.13)	0.88 (0.53 , 1.47)	0.72 (0.41 , 1.25)	0.96 (0.61 , 1.52)	0.86 (0.46 , 1.59)	1.15 (0.57 , 2.30)	1.07 (0.69 , 1.66)	1.18 (0.66 , 2.12)	0.96 (0.49 , 1.88)
MMP	Q1	Ref	Ref	Ref	Ref	Ref	Ref	Ref	Ref	Ref	Ref	Ref	Ref	Ref	Ref	Ref
	Q2	0.85 (0.53 , 1.36)	0.77 (0.44 , 1.34)	0.91 (0.30 , 2.77)	1.01 (0.62 , 1.67)	1.48 (0.71 , 3.08)	0.61 (0.29 , 1.31)	0.93 (0.63 , 1.38)	0.94 (0.57 , 1.55)	0.61 (0.28 , 1.34)	1.12 (0.69 , 1.83)	1.14 (0.63 , 2.06)	2.13 (0.59 , 7.68)	0.89 (0.57 , 1.39)	1.09 (0.62 , 1.91)	0.47 (0.21 , 1.05)#
	Q3	0.92 (0.57 , 1.49)	0.96 (0.55 , 1.68)	0.89 (0.28 , 2.77)	1.17 (0.71 , 1.93)	1.37 (0.64 , 2.94)	0.81 (0.38 , 1.73)	1.00 (0.68 , 1.48)	1.14 (0.69 , 1.89)	0.60 (0.27 , 1.32)	1.43 (0.88 , 2.32)	1.10 (0.60 , 2.01)	3.11 (0.88 , 11.04)#	1.00 (0.65 , 1.56)	1.45 (0.84 , 2.53)	0.47 (0.21 , 1.04)#
	Q4	1.25 (0.77 , 2.04)	1.15 (0.63 , 2.09)	1.42 (0.46 , 4.38)	1.02 (0.61 , 1.73)	1.49 (0.66 , 3.34)	0.64 (0.30 , 1.40)	0.98 (0.65 , 1.49)	1.21 (0.70 , 2.10)	0.53 (0.24 , 1.20)	1.42 (0.85 , 2.37)	1.32 (0.69 , 2.52)	2.73 (0.76 , 9.83)	0.75 (0.46 , 1.21)	0.81 (0.42 , 1.55)	0.38 (0.17 , 0.88)*
MiNP	Q1	Ref	Ref	Ref	Ref	Ref	Ref	Ref	Ref	Ref	Ref	Ref	Ref	Ref	Ref	Ref
	Q2	0.76 (0.35 , 1.65)	0.65 (0.15 , 2.84)	0.90 (0.35 , 2.29)	1.00 (0.54 , 1.85)	0.34 (0.05 , 2.19)	1.15 (0.59 , 2.22)	0.98 (0.55 , 1.77)	0.84 (0.22 , 3.21)	0.98 (0.50 , 1.90)	0.67 (0.32 , 1.40)	0.87 (0.16 , 4.83)	0.64 (0.28 , 1.50)	1.74 (0.85 , 3.56)	0.57 (0.11 , 2.85)	2.20 (0.96 , 5.01)#
	Q3	0.91 (0.33 , 2.54)	0.89 (0.12 , 6.48)	1.03 (0.30 , 3.51)	1.22 (0.53 , 2.81)	0.30 (0.02 , 3.82)	1.44 (0.59 , 3.51)	0.98 (0.45 , 2.16)	0.63 (0.10 , 4.08)	1.01 (0.41 , 2.48)	0.77 (0.29 , 2.06)	2.00 (0.22 , 18.52)	0.62 (0.20 , 1.96)	3.68 (1.45 , 9.35)*	0.75 (0.08 , 6.95)	5.05 (1.75 , 14.58)*
	Q4	0.97 (0.25 , 3.77)	1.00 (0.07 , 15.07)	1.11 (0.22 , 5.52)	1.44 (0.47 , 4.35)	0.12 (0.00 , 4.74)	1.90 (0.58 , 6.20)	1.10 (0.38 , 3.17)	0.60 (0.05 , 7.92)	1.18 (0.36 , 3.86)	0.71 (0.19 , 2.66)	1.81 (0.09 , 36.90)	0.55 (0.12 , 2.61)	2.80 (0.81 , 9.70)	0.33 (0.01 , 7.52)	4.14 (1.03 , 16.69)*
ΣHMW	Q1	Ref	Ref	Ref	Ref	Ref	Ref	Ref	Ref	Ref	Ref	Ref	Ref	Ref	Ref	Ref
	Q2	1.14 (0.72 , 1.80)	1.07 (0.65 , 1.78)	2.54 (0.52 , 12.39)	0.98 (0.59 , 1.63)	1.00 (0.52 , 1.92)	0.89 (0.36 , 2.17)	0.93 (0.63 , 1.38)	0.81 (0.51 , 1.29)	1.76 (0.71 , 4.38)	0.54 (0.33 , 0.88)*	0.47 (0.26 , 0.85)*	0.86 (0.28 , 2.65)	1.00 (0.65 , 1.55)	0.99 (0.59 , 1.65)	1.09 (0.40 , 2.95)
	Q3	1.16 (0.71 , 1.87)	1.15 (0.65 , 2.05)	2.36 (0.50 , 11.09)	0.72 (0.43 , 1.22)	0.53 (0.22 , 1.28)	0.72 (0.31 , 1.69)	1.01 (0.67 , 1.52)	0.95 (0.56 , 1.61)	1.71 (0.71 , 4.10)	0.55 (0.33 , 0.91)*	0.49 (0.25 , 0.97)*	0.77 (0.26 , 2.28)	0.76 (0.48 , 1.21)	0.82 (0.45 , 1.50)	0.77 (0.29 , 2.02)
	Q4	0.81 (0.48 , 1.38)	0.60 (0.29 , 1.25)	1.94 (0.41 , 9.10)	0.72 (0.43 , 1.23)	0.83 (0.34 , 2.04)	0.68 (0.29 , 1.59)	0.85 (0.56 , 1.30)	0.77 (0.42 , 1.43)	1.43 (0.60 , 3.40)	0.92 (0.56 , 1.50)	1.07 (0.55 , 2.08)	1.14 (0.40 , 3.27)	0.78 (0.48 , 1.27)	0.74 (0.36 , 1.52)	0.84 (0.32 , 2.18)
ΣLMW	Q1	Ref	Ref	Ref	Ref	Ref	Ref	Ref	Ref	Ref	Ref	Ref	Ref	Ref	Ref	Ref
	Q2	1.22 (0.78 , 1.92)	1.07 (0.60 , 1.88)	1.60 (0.72 , 3.56)	1.37 (0.88 , 2.11)	1.52 (0.70 , 3.28)	1.26 (0.74 , 2.14)	0.85 (0.59 , 1.22)	0.90 (0.54 , 1.51)	0.75 (0.44 , 1.28)	1.23 (0.79 , 1.92)	1.36 (0.75 , 2.48)	1.24 (0.62 , 2.45)	1.24 (0.82 , 1.89)	1.71 (0.97 , 3.02)#	0.89 (0.48 , 1.65)
	Q3	1.11 (0.70 , 1.77)	0.99 (0.56 , 1.77)	1.41 (0.62 , 3.21)	1.28 (0.83 , 2.00)	1.20 (0.53 , 2.69)	1.30 (0.76 , 2.23)	0.94 (0.65 , 1.36)	0.88 (0.52 , 1.49)	0.94 (0.54 , 1.63)	0.94 (0.59 , 1.50)	0.87 (0.45 , 1.67)	1.08 (0.53 , 2.18)	1.06 (0.69 , 1.63)	1.23 (0.68 , 2.23)	0.86 (0.45 , 1.61)
	Q4	0.98 (0.62 , 1.55)	0.89 (0.51 , 1.55)	1.20 (0.51 , 2.80)	1.48 (0.96 , 2.29)#	1.63 (0.77 , 3.44)	1.44 (0.84 , 2.47)	0.71 (0.50 , 1.03)#	0.86 (0.52 , 1.43)	0.58 (0.34 , 1.01)#	1.19 (0.76 , 1.85)	1.02 (0.56 , 1.86)	1.47 (0.74 , 2.91)	1.03 (0.68 , 1.58)	0.84 (0.46 , 1.54)	1.16 (0.63 , 2.15)
ΣDEHP	Q1	Ref	Ref	Ref	Ref	Ref	Ref	Ref	Ref	Ref	Ref	Ref	Ref	Ref	Ref	Ref
	Q2	0.90 (0.57 , 1.43)	0.84 (0.49 , 1.42)	1.47 (0.44 , 4.89)	0.82 (0.50 , 1.34)	0.83 (0.42 , 1.66)	0.82 (0.37 , 1.80)	0.90 (0.61 , 1.33)	0.89 (0.55 , 1.43)	1.23 (0.55 , 2.73)	0.81 (0.51 , 1.30)	0.67 (0.37 , 1.20)	1.77 (0.56 , 5.63)	1.11 (0.72 , 1.72)	1.25 (0.74 , 2.11)	1.05 (0.42 , 2.64)
	Q3	1.25 (0.79 , 1.98)	1.27 (0.74 , 2.19)	1.81 (0.57 , 5.79)	0.74 (0.45 , 1.21)	0.56 (0.25 , 1.25)	0.79 (0.37 , 1.70)	1.06 (0.72 , 1.57)	0.89 (0.53 , 1.48)	1.67 (0.76 , 3.65)	0.71 (0.43 , 1.15)	0.54 (0.28 , 1.04)#	1.52 (0.49 , 4.76)	0.96 (0.61 , 1.50)	0.88 (0.49 , 1.58)	1.13 (0.46 , 2.76)
	Q4	0.69 (0.41 , 1.15)	0.60 (0.30 , 1.20)	1.05 (0.32 , 3.42)	0.66 (0.40 , 1.09)	0.68 (0.29 , 1.60)	0.67 (0.32 , 1.44)	0.86 (0.57 , 1.30)	0.78 (0.44 , 1.40)	1.21 (0.56 , 2.61)	1.11 (0.69 , 1.80)	1.15 (0.61 , 2.19)	2.04 (0.67 , 6.23)	0.88 (0.55 , 1.42)	0.78 (0.39 , 1.54)	1.00 (0.42 , 2.43)
ΣDBP	Q1	Ref	Ref	Ref	Ref	Ref	Ref	Ref	Ref	Ref	Ref	Ref	Ref	Ref	Ref	Ref
	Q2	0.94 (0.60 , 1.47)	0.83 (0.47 , 1.47)	1.06 (0.51 , 2.21)	1.21 (0.80 , 1.84)	2.11 (0.95 , 4.69)#	0.93 (0.56 , 1.54)	1.01 (0.71 , 1.43)	0.90 (0.55 , 1.50)	1.06 (0.63 , 1.79)	1.05 (0.68 , 1.62)	0.94 (0.51 , 1.73)	1.20 (0.64 , 2.28)	1.32 (0.87 , 1.99)	1.88 (1.06 , 3.34)*	0.89 (0.49 , 1.64)
	Q3	1.14 (0.73 , 1.78)	1.21 (0.69 , 2.13)	1.05 (0.49 , 2.23)	1.29 (0.84 , 1.98)	2.12 (0.93 , 4.83)#	1.02 (0.60 , 1.72)	0.74 (0.51 , 1.06)	0.93 (0.55 , 1.57)	0.62 (0.36 , 1.06)#	1.01 (0.65 , 1.58)	1.13 (0.62 , 2.07)	0.98 (0.50 , 1.93)	1.28 (0.83 , 1.95)	1.34 (0.73 , 2.47)	1.06 (0.58 , 1.95)
	Q4	0.85 (0.53 , 1.35)	0.96 (0.55 , 1.67)	0.62 (0.26 , 1.48)	1.28 (0.82 , 1.99)	2.31 (1.05 , 5.08)*	0.94 (0.54 , 1.64)	0.77 (0.53 , 1.12)	0.74 (0.44 , 1.23)	0.82 (0.47 , 1.44)	0.89 (0.56 , 1.41)	0.78 (0.42 , 1.44)	1.12 (0.55 , 2.26)	1.27 (0.82 , 1.95)	1.39 (0.77 , 2.50)	1.11 (0.59 , 2.12)

Abbreviations: MEHP (Mono-ethylhexyl phthalate); MEOHP (Mono-2-ethyl-5-oxohexyl phthalate); MEHHP (Mono-2-ethyl-5-hydroxylhexyl phthalate); MECPP (methylerythritol cyclodiphosphate); MCMHP (Mono(2-carboxymethylhexyl) phthalate); MBzP (monobenzyl phthalate);MEP (monoethyl phthalate); MiBP (monoisobutyl phthalate); MnBP (mono-n-butyl phthalate); MMP (Mono-methyl phthalate); MiNP (Mono-isooctyl phthalate); ΣHMW (high molecular weight) = (Mono-ethylhexyl phthalate (MEHP) + Mono-2-ethyl-5-oxohexyl phthalate (MEOHP) + Mono-2-ethyl-5-hydroxylhexyl phthalate (MEHHP) + Mono-benzyl phthalate (MBzP) + Mono-isooctyl phthalate (MiNP). ΣLMW (low molecular weight) = Mono-ethyl phthalate (MEP) + Mono-isobutyl phthalate (MiBP) + Mono-n-butyl phthalate (MnBP) + Mono-methyl phthalate (MMP). ∑DEHP (di(2-ethylhexyl) phthalate [molar sum of mono-(2-ethylhexyl) phthalate (MEHP), mono-(2-ethyl-5oxohexyl) phthalate (MEOHP), and mono-(2-ethyl-5-hydroxyhexyl) phthalate (MEHHP), and mono-(2-ethyl-5-carboxypentyl) phthalate (MECPP) , and Mono(2-carboxymethylhexyl) phthalate (MCMHP]). ΣDBP (dibutyl phthalate) = Mono-isobutyl phthalate (MiBP) + Mono-n-butyl phthalate (MnBP).

[#] $P < 0.10$ *$P < 0.05$.

Adjusted for urinary creatinine, age, sex, education, and working status.

Supplemental Table 2 Prevalence odds ratios for the association between urinary metabolites concentrations and metabolic syndrome stratified by menopausal status in females.

Phthalate Metabolites		Females (n=644)			
		premenopausal (n=354)		postmenopausal (n=290)	
MEOHP					
	Q1	Ref		Ref	
	Q2	0.35	(0.12, 1.06) #	1.06	(0.38, 2.96)
	Q3	0.73	(0.29, 1.86)	1.49	(0.58, 3.86)
	Q4	0.48	(0.18, 1.29)	0.60	(0.21, 1.72)
MEHHP					
	Q1	Ref		Ref	
	Q2	0.51	(0.18, 1.42)	2.09	(0.80, 5.50)
	Q3	0.45	(0.17, 1.16) #	1.34	(0.52, 3.46)
	Q4	0.82	(0.32, 2.06)	1.05	(0.39, 2.79)
MBzP					
	Q1	Ref		Ref	
	Q2	0.09	(0.02, 0.39)*	1.28	(0.52, 3.13)
	Q3	0.47	(0.19, 1.13) #	1.53	(0.58, 4.05)
	Q4	0.43	(0.17, 1.09) #	0.78	(0.27, 2.23)
MiNP					
	Q1	Ref		Ref	
	Q2	2.44	(0.65, 9.25)	1.58	(0.47, 5.38)
	Q3	4.10	(0.78, 21.65) #	1.90	(0.36, 10.05)
	Q4	4.11	(0.50 34.06)	1.92	(0.20, 18.39)
ΣDEHP					
	Q1	Ref		Ref	
	Q2	0.71	(0.17, 2.88)	4.67	(0.55, 40.10)
	Q3	0.49	(0.12, 1.96)	9.58	(1.18, 77.75)*
	Q4	0.63	(0.16, 2.43)	5.06	(0.62, 41.34)

Abbreviations: MEOHP (Mono-2-ethyl-5-oxohexyl phthalate); MEHHP (Mono-2-ethyl-5-hydroxylhexyl phthalate); MBzP (monobenzyl phthalate); MiNP (Mono-isooctyl phthalate); ∑DEHP (di(2-ethylhexyl) phthalate [molar sum of mono-(2-ethylhexyl) phthalate (MEHP), mono-(2-ethyl-5oxohexyl) phthalate (MEOHP), and mono-(2-ethyl-5-hydroxyhexyl) phthalate (MEHHP), and mono-(2-ethyl-5-carboxypentyl) phthalate (MECPP) , and Mono(2-carboxymethylhexyl) phthalate (MCMHP]).

$^{\#}P < 0.10$ $^{*}P < 0.05$.

Adjusted for urinary creatinine, age, education, and working status
